# Skeletal muscle dedifferentiation requires centrosomes

**DOI:** 10.1038/s41536-026-00509-3

**Published:** 2026-09-25

**Authors:** Elaiyaraja Subramanian, Anoop Kumar, Ketan Mishra, Gonçalo Brito, Matthew Kirkham, András Simon

**Affiliations:** Department of Cell and Molecular Biology, Karolinska Institutet, Stockholm, Sweden

**Keywords:** Cell biology, Developmental biology

## Abstract

Multinucleated skeletal muscle cells are stably withdrawn from the cell cycle in most vertebrates. Muscle dedifferentiation, however, naturally occurs during limb regeneration in newts and can be experimentally induced in mammalian myotubes. Here we addressed the dynamics of centrosomes, which are key organelles for cell proliferation during myogenic differentiation and dedifferentiation in a cross-species comparative setting. We show that, unlike their mammalian counterparts, newt muscle cells retain centrosomes during differentiation, and their abrogation during regeneration interferes with myogenic dedifferentiation as well as blastema formation in newts. Mammalian myotubes, which are experimentally induced to dedifferentiate, give rise to progeny that regain centrosomes through a process that depends on inhibition of the tumor suppressor, p53. We also find that regulation of the subcellular localization of Polo-Like Kinase 4, rather than its expression level, is a hallmark of myogenic differentiation and dedifferentiation, revealing a novel cellular process underlying the plasticity of the differentiated state.

## Introduction

Skeletal muscle differentiation involves stable withdrawal from the cell cycle, which is generally referred to as the postmitotic state^1–3^. Reversal of myogenic differentiation occurs naturally during limb regeneration and is characterized by three main phases: (i) fragmentation of the syncytium, (ii) temporal downregulation of a select set of muscle differentiation markers in the myofiber-derived mononucleate progeny, (iii) which subsequently re-enter the cell cycle and proliferate^4–7^. While muscle dedifferentiation in newts has been well described at the cellular level^8,9^ and some of its inductive molecular underpinnings identified^5,6,10–12^, the dynamics of centrosomes with microtubule organizing capacity, which are key cellular organelles for cell division^13–17^, have remained unexplored. In mammals, centrosomes are lost during muscle differentiation^18–21^, and microtubule organization shifts to non-centrosomal locations^21–23^. Hence, three principal scenarios are possible in newts: (i) differentiated muscle cells could retain centrosomes in newts; (ii) centrosomes could be lost, similarly to mammals, but assembled de novo because of or at least concomitant with, dedifferentiation; (iii) myofiber-derived mononucleate cells could regain proliferative potential without centrosomes. Although being a rare phenomenon, cell proliferation without centrosomes has been described in certain contexts, like development of fruit flies and mice^24,25^, and at whole organism level in asexually reproducing flatworms^26^.

In contrast to newts, skeletal muscle dedifferentiation does not occur naturally in mammals^27,28^. Nevertheless, dedifferentiation is possible to induce experimentally in cultures of myotubes by myoseverin, which causes microtubule depolymerization and fragmentation of myotubes into mononucleate cells^29^. Rigorous cell tracking experiments demonstrated that myoseverin-induced myotube fragmentation produces non-proliferative mononucleate cells^30^ because of the initiation of an apoptotic program^6^. Importantly, inhibition of both caspases^25^ as well as the tumor suppressor, p53, in combination with myoseverin treatment is required for myotube-derived mononucleate cells to become proliferative^6^. Upon expansion in culture, these cells are able to contribute to muscle regeneration in mice^6^.

Here we used this experimentally tractable cell-based assay system to ask if centrosomes become regenerated in the myotube-derived mammalian mononucleate progeny as these cells reenter the cell cycle and reach S-phase. We show that centrosomes become detectable in dedifferentiated cells only if p53 is inhibited. Furthermore, we identify that the expression level of a key regulator of centrosome assembly, polo-like kinase 4 (PLK4)^31,32^ remains stable during muscle differentiation. Instead of a change in expression level, PLK4 shifts its subcellular localization during myogenic differentiation. We also show that newt muscle cells retain their centrosomes with microtubule-organizing activity in their differentiated state, and that inhibition of PLK4 interferes with myogenic dedifferentiation during limb regeneration. Collectively, these data provide both an explanation for why newt, but not mammalian muscle gives rise to proliferating progeny after an injury, as well as define a link between p53 and the dynamics of PLK4 subcellular localization in the context of muscle dedifferentiation.

## Results

### Centrosome organization in differentiating muscle cells

**Fig. 1:**
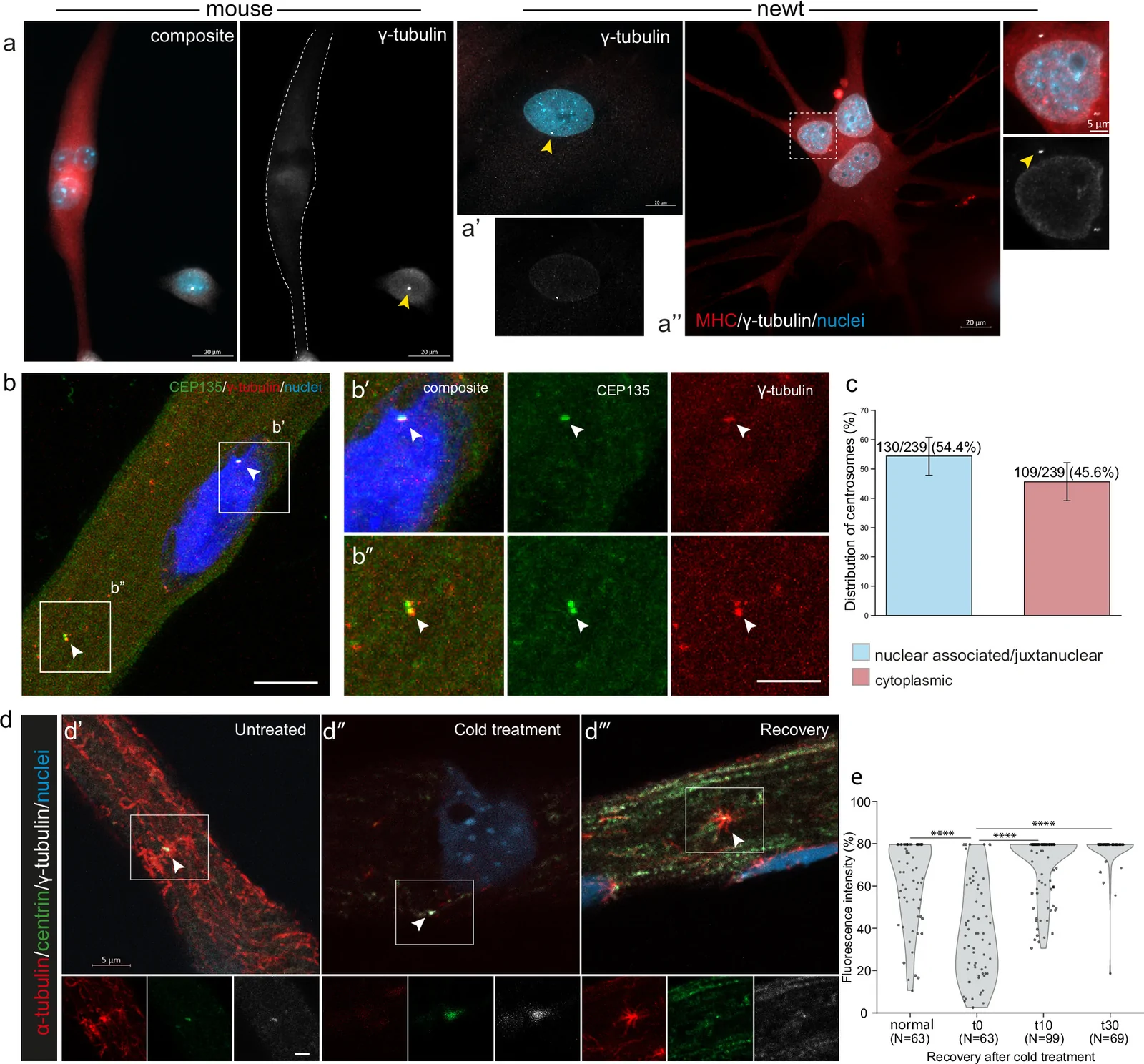
In contrast to mouse muscle, differentiated newt muscle cells retain centrosomes. **a-a”** Differentiated mouse and newt muscle cells in culture. **a** A differentiated mouse myotube without centrosomes (right panel, outlined with white dotted line). Note the centrosome in the adjacent mononucleate cell (right panel, arrowed yellow). **a’** A newt mononucleate cell with a centrosome (arrowed yellow). **a”** Multinucleate newt myotube showing centrosomes (inset, enlarged in the right panel). **b** An area of a newt myofiber showing immunostaining of centrosomes with two markers, CEP135 and γ-tubulin. **b’, b”** Magnified area showing co-immunostaining with antibodies to centrosomes (arrowed white). **c** Association of centrosomes with nuclei or along the myofiber cytoplasm. Centrosome location varies in myofibers, with 54.4% of the centrosomes being associated with myonuclei (juxtanuclear, **b’**) and the remaining 45.6% being located along the myofiber cytoplasm (distant, **b”**). A total of 239 centrosomes were analyzed from newt myofibers pooled from the forelimbs (*n* = 6). Error bars represent 95% confidence intervals using the exact method**. d-d”’** Microtubule regrowth assay. The microtubules were depolymerized through cold treatment on ice for three hours and subsequently allowed to recover at normal temperature. After 10 seconds, recovery and repolymerization of microtubules occur from the centrosomes. Lower panels show corresponding areas magnified from the upper panel. **e** Quantification of mean intensity from α-tubulin during microtubule regrowth assay. The amount of α-tubulin is significantly reduced after cold treatment and rapidly restored upon recovery. Normal myofibers; t0, 3 h after cold treatment; t10, 10 seconds after recovery; t30. Violin plot represents individual cells (one-way ANOVA with Kruskal-Wallis test, **** = *p* < 0.0001). Pooled myofiber cultures from the forelimbs (*n* = 6). Scale bars: **a**-**a”** 20 µm, **b** 10 µm, **b’, b”** 2 µm, **d** 5 µm, inset 2 µm.

We compared mouse and newt myogenic cells which were stimulated to undergo differentiation, resulting in the generation of multinucleate myotubes expressing the differentiation marker myosin heavy chain (MHC) (Fig. 1a-a”). These differentiation events are well-documented, and the myotubes attain stable withdrawal from the cell cycle in both mouse and newt myotubes^3,7,10,33^. While we found that mouse myotubes gradually lost centrosomes (Fig. 1a), newt myotubes retained centrosomes indicated by canonical γ-tubulin localization (Fig. 1a’-a”). Having established that newt myotubes differ from their mammalian counterparts in terms of the presence of centrosomes, we next asked whether these features are retained in newt myofibers freshly isolated from mature limbs (Fig. S1a–c). We evaluated the presence of centrosomes in myofibers by analyzing the expression of γ-tubulin and CEP135. We found expression and colocalization of these two markers, with 54.4% of myonuclei in a classical nuclear associated/juxtanuclear position, while the remaining centrosomal foci (45.6%) were positioned away from the nuclei in myofibers (Fig. 1b-b”, c).

Next, we assessed whether centrosomes within the myofibers were active by evaluating their microtubule organizing activity (MTOC assay)^34^. We performed a microtubule regrowth assay using cold microtubule depolymerization and allowing their subsequent regrowth for a short interval at physiological temperature. We found that cold treatment efficiently depleted their microtubules, and rapid recovery occurred upon shifting the treated cells to physiological temperature (Fig. 1d-d”’). These microtubules emanated from centrosomes that expressed both centrin and γ-tubulin (Fig. 1d”). To validate the microtubule organizing activity of centrosomes in myofibers, we quantified the intensity of α-tubulin during microtubule regrowth. The intensity was significantly reduced upon cold treatment and subsequently restored to normal at physiological temperature (Fig. 1e). Taken together, we conclude that differentiated newt muscle cells differ from their mammalian counterparts by retaining an active state of centrosome components that can serve as microtubule organizing centers.

### Centrosomes are retained in dedifferentiated muscle-derived newt limb blastemal cells

**Fig. 2:**
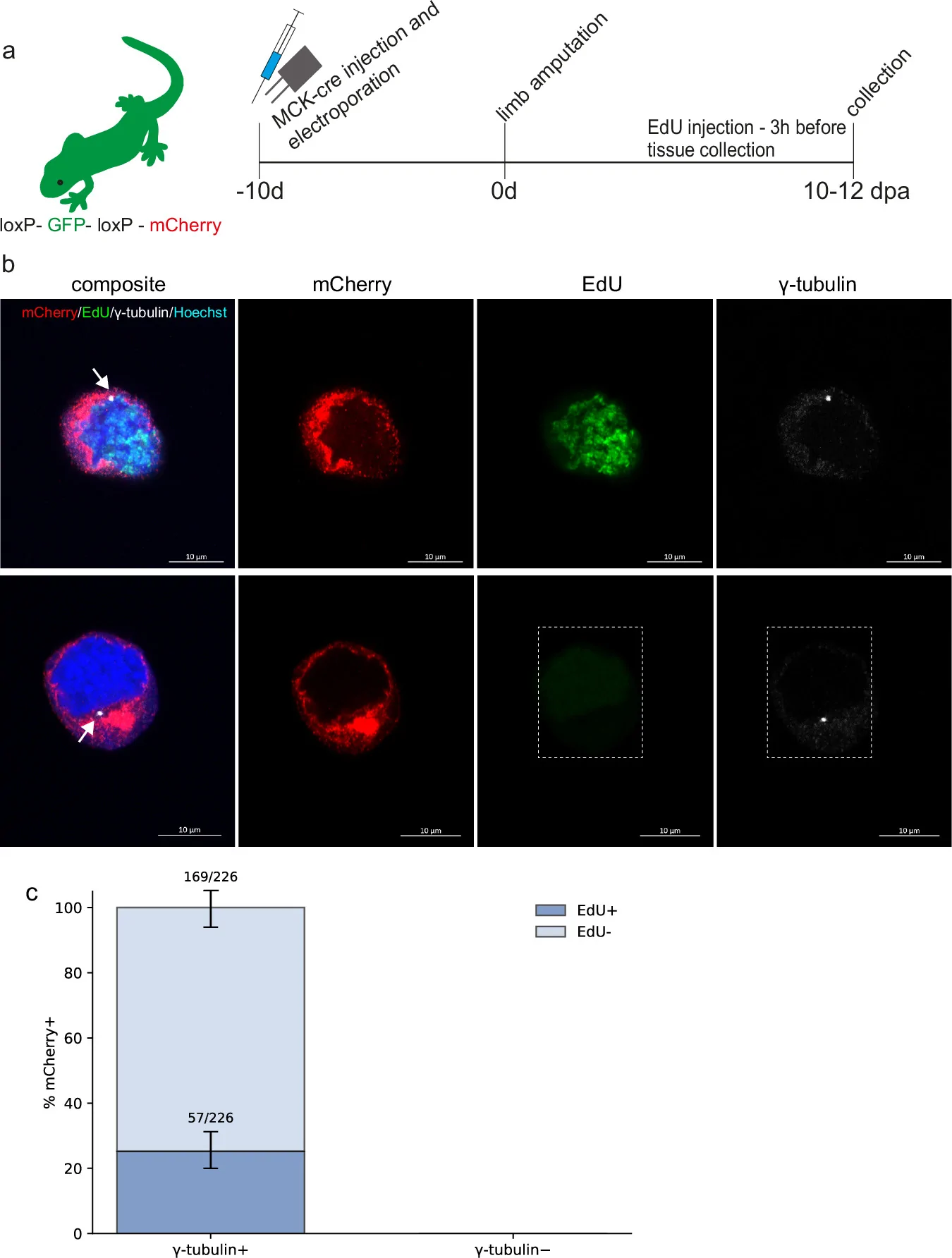
Centrosomes are retained by dedifferentiated mononucleate cells during newt limb regeneration. **a** Outline of the experiment illustrating Cre-induced recombination leading to mCherry expression in myfibers. **b** Dedifferentiated myofiber-derived mCherry+ cells isolated from limb blastemas by the Cytospin method. Immunostaining showing EdU+ (upper panel) and EdU- (lower panel) mCherry+ cells with γ-tubulin+ centrosomes (arrowed, white). **c** Distribution of centrosomes in mCherry+ cells. Note the absence of γ-tubulin- cells. Error bars represent 95% confidence intervals (Wilson method). Five limb blastemas were pooled for isolation of cells/cytospin assay in two independent assays. Scale bar, 10 μm.

Newt myofibers show remarkable cellular plasticity and dedifferentiate to generate proliferating mononucleate cells during limb regeneration. This process has been demonstrated using several cell tracking strategies, including transgenic reporter lines^4,5,10^. However, whether the resultant mononucleate progeny cells have centrosomes is unclear. Therefore, we used a newt transgenic reporter line (loxP-GFP-loxP-mCherry)^5,35,36^ to evaluate the possible inheritance of centrosomes in myofiber-derived cells. Upon muscle-specific Cre expression and resultant recombination, myofibers and their progeny cells become permanently labeled with mCherry. A schematic of the experimental procedures is outlined in Fig. 2a. Cre was induced by focal electroporation into the right limb, which resulted in recombination (Fig. S2a, b, b’), while no recombination events were observed in the contralateral control limb (Fig. S2c, c’). The nucleotide analog, EdU, was injected to capture S-phase reentry by myofiber-derived progeny cells during blastema formation. Mid-bud limb blastemas were collected and enzymatically dissociated into single cells (for details, see Methods). The isolated single cells were captured onto Cytospin slides and processed for immunofluorescence. We identified mCherry + /EdU + /γ-tubulin+ cells (Fig. 2b, upper panel) and mCherry + /EdU−/γ-tubulin+ cells (Fig. 2b, lower panel) derived by dedifferentiation of muscle fibers during the regenerative process. The percentage distribution of these populations is depicted in Fig. 2c which shows S-phase reentry and formation of centrosomes in muscle-derived progeny cells. Importantly, all mCherry+ (i.e. myofiber-derived) cells had centrosomes, indicated by canonical γ-tubulin staining.

To further validate the retention of centrosomes in myofiber-progeny cells during muscle dedifferentiation, we performed in vivo cell tracking experiments using the strategy described above (Figs. 2a and S3a). We evaluated serial longitudinal sections of the limb blastema after immunostaining. We detected mCherry + /EdU + /γ-tubulin+ cells in the blastema (Fig. S3b). To corroborate that myofiber-derived cells contained centrosomes, we used an alternative Cre-inducible reporter line (loxP-mCherry-loxP-H2B:YFP), which resulted in the appearance of nYFP + /EdU + /γ-tubulin+ cells in the limb blastema (Fig. S4). Thus, we conclude that both myofibers and their dedifferentiated blastemal progeny retain centrosomes in the newt limb.

### Reassembly of centrosomes during dedifferentiation of mouse myotubes

**Fig. 3:**
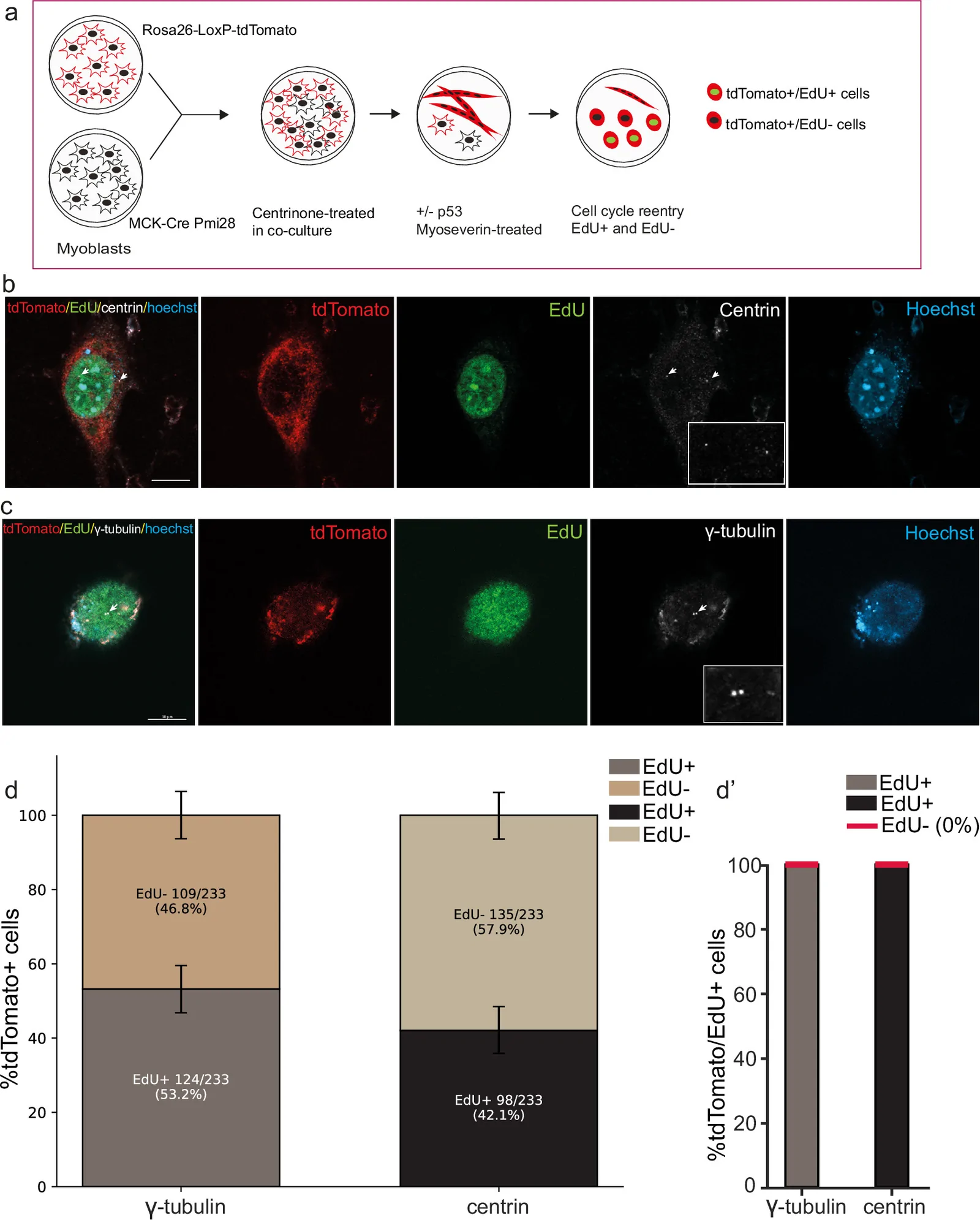
p53-inhibition-dependent reassembly of centrosomes during mouse muscle dedifferentiation. **a** Schematic showing the assay protocol. Rosa26 myoblasts (CAG:loxP-stop-loxP:tdTomato) and Pmi28 myoblasts (MCK-Cre) were cultured separately in the presence of centrinone for four days. The cells were plated together at a 3:1 ratio in differentiation medium in the presence of centrinone. During the fusion stage, on day 5, the cultures were treated with a p53 inhibitor for 24 h. Cellularisation was induced by treatment with myoseverin for 24 h. Thereafter, the medium was changed to growth medium containing direct and indirect caspase inhibitors, growth factors, and pulsed with EdU. **b** A dedifferentiated mononucleate cell showing S-phase reentry and immunoreactivity with centrin. **c** A dedifferentiated cell showing S-phase reentry and immunoreactivity with γ-tubulin. **d**, **d’** Distribution of centrosome markers and S-phase reentry in dedifferentiated myotube-derived mononucleate cells in the presence of p53 inhibitor (culture plates, *n* = 8 in each assay). Note that all EdU+ cells are also γ-tubulin+ and centrin+. Error bars represent 95% confidence intervals (Wilson method). Scale bar, 10 μm; inset, 0.5 μm.

To test whether experimentally induced dedifferentiation of mouse myotubes leads to reassembly of centrosomes in the mononucleate progeny, we optimized the protocol by Wang, 2015^6^. A schematic drawing of the assay protocol is shown in Fig. 3a (for details, see Methods). In brief, cellularization of myotubes was induced by myoseverin treatment, and subsequent cell cycle reentry of the resultant mononucleate, myotube-derived cells was achieved by inhibition of p53 using Pifithrin-α^37,38^. For specifically tracing myotube-derived cells, we fused primary myoblasts carrying a reporter which encodes conditional expression of tdTomato with myoblasts expressing Cre-recombinase under the control of the muscle creatine kinase (MCK) promoter.

To ensure that no residual centrosomes were present in the myotubes before the dedifferentiation assay, we treated myoblasts with centrinone, which eliminates centrosomes^39,40^. Based on γ-tubulin staining, 99.4% of the myotubes thus formed lacked centrosomes in the cultures (Fig. S5a, a’). In accordance with previous data^6^, cellularization upon myoseverin treatment occurred irrespective of p53-inhibition. Myoseverin treatment without p53 inhibition resulted in the generation of non-proliferative tdTomato+ mononucleate cells as determined by EdU incorporation (Fig. S5b).

To evaluate the possible reassembly of centrosomes in myotube-derived, mononucleate progeny, we analyzed the expression of two different centrosome markers, centrin and γ-tubulin. These analyses revealed myotube-derived tdTomato+/EdU+/centrin+ as well as tdTomato+/EdU+/ γ-tubulin+ cells, indicating the reassembly of centrosomes following myogenic dedifferentiation (Fig. 3b, c). In independent, parallel assays, we found that 53.2% γ-tubulin+ and 42.1% centrin+ cells had incorporated EdU (Fig. 3d). Thus, a substantial portion of centrosome-containing cells were also EdU– (Fig. 3d), similar to the observations in the dedifferentiated mononucleate cells in the newt limb blastema. This population may represent cells in which S-phase did not coincide with the EdU-pulsing. Since all tdTomato+/EdU+ cells had centrosomes (Fig. 3d’), the data collectively indicate that reassembly of centrosomes is critical to generate proliferative mononucleate progeny in a p53-inhibition-dependent manner during muscle dedifferentiation.

### Dynamic subcellular relocalization of PLK4 during muscle differentiation and dedifferentiation

Disassembly of the centrosomes during muscle differentiation and their reappearance in myotube-derived progeny prompted us to examine the status of PLK4, a key regulator of centrosome function^32^, during muscle differentiation and dedifferentiation.

**Fig. 4:**
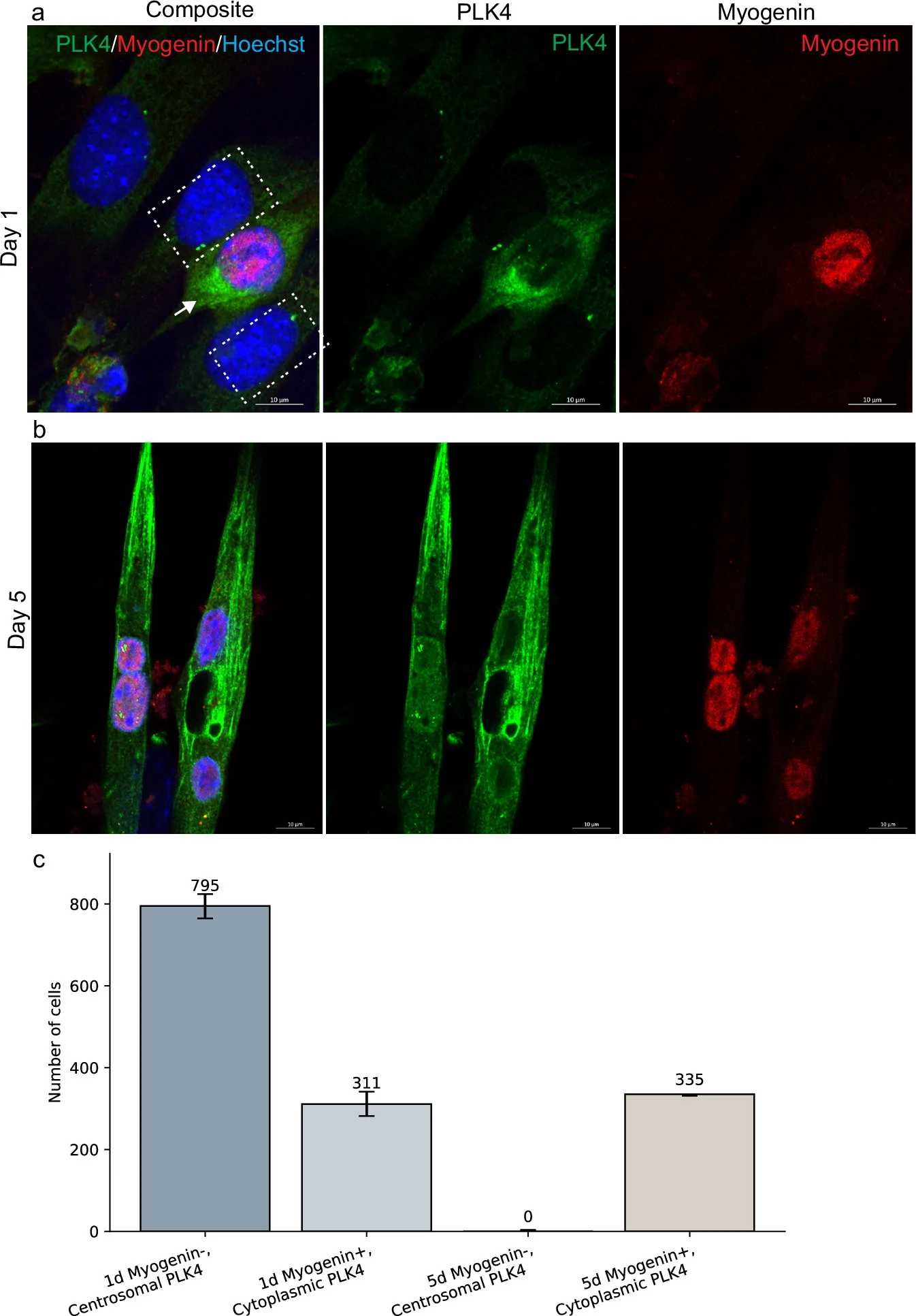
Progressive redistribution of PLK4 in mouse muscle cells by differentiation. **a** Mouse myoblasts on Day1 of induced differentiation in low serum. Note the redistribution of PLK4 to cytoplasm in a myogenin+ cell (arrowed white), while adjacent myogenin– myoblasts (boxed, dotted white) show centrosomal PLK4. **b** Day 5 of differentiation; in myogenin+ myotubes PLK4 is localized to a cytoplasmic pool. **c** Distribution of centrosomal/cytoplasmic PLK4 in myogenin+/− cells. Error bars represent 95% confidence intervals using the exact method (pooled culture plates, *n* = 3). Scale bars, 10 μm.

We found that PLK4 was localized to the centrosomes in mouse myoblasts (Fig. S6), and notably we did not find any γ-tubulin+/PLK4− myoblasts in the assay (Fig. S6). Additionally, incorporation of EdU indicated that centrosomal localization of PLK4 was not dependent on cell cycle stages in myoblasts, since both EdU+ and EdU− myoblasts showed the same localization pattern (Fig. S7). Upon induced differentiation of myoblasts, a shift of PLK4 localization occurred from the centrosomes, appearing throughout the cytoplasmic compartment. Relocalization of PLK4 was not dependent on cell fusion but rather on induction of differentiation, as indicated by cytoplasmic localization in myogenin-expressing mononucleate cells (Fig. 4a, c). Myogenin– mononucleate cells displayed canonical centrosomal localization of PLK4 (Fig. 4a, c). In fully differentiated myotubes that express both myogenin and myosin heavy chain, PLK4 was distributed throughout the cell, extending to cytoplasmic and myofibrillar compartments (Figs. 4b, c and S8).

**Fig. 5:**
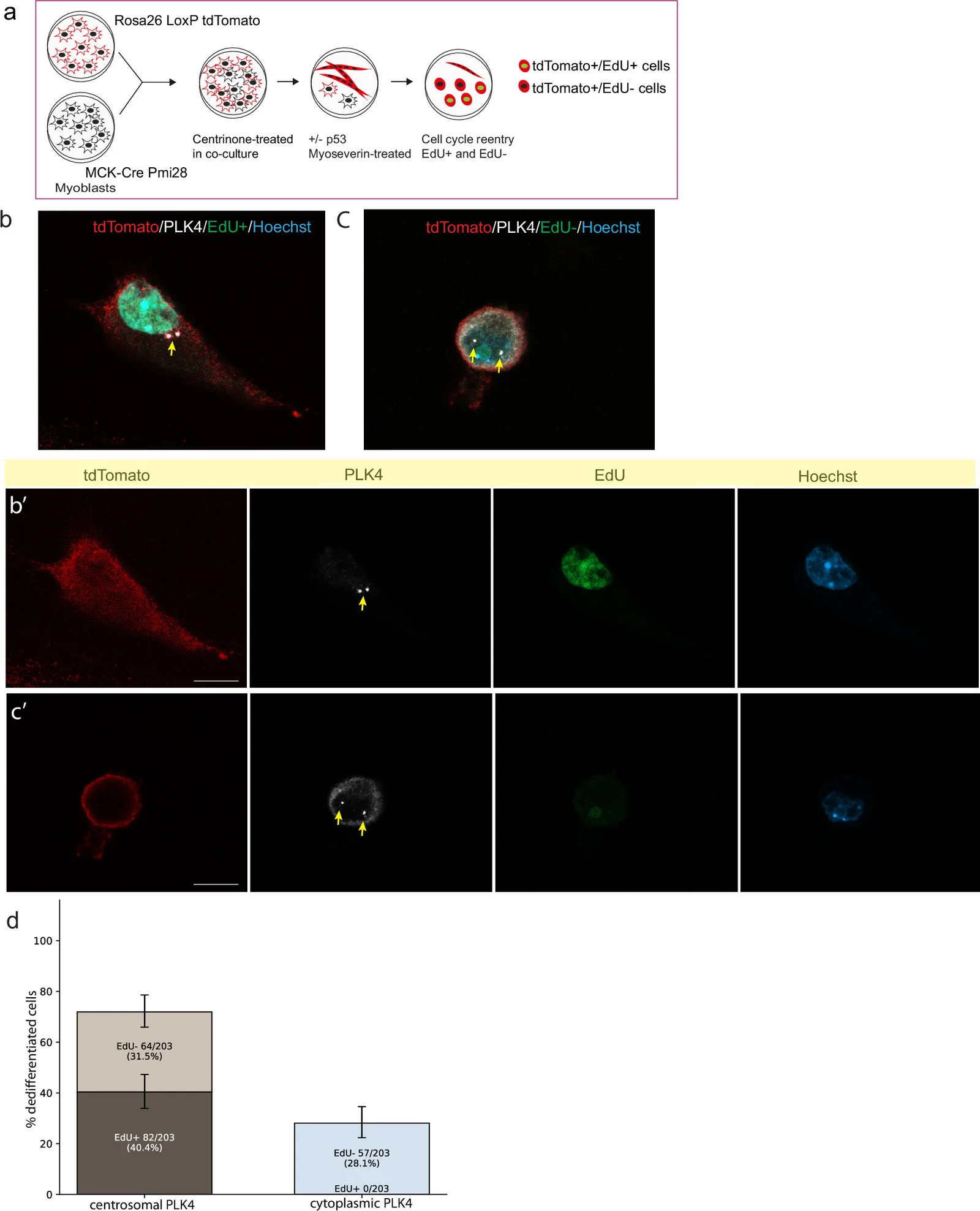
Centrosomal relocalisation of PLK4 after cellularization of mouse myotubes. **a** Schematic showing the assay protocol. **b**, **c** Examples of EdU+ and EdU– myotube-derived mononucleate cell. **b’, c’**. Split channels showing the four different labels in the cells shown in (**b**, **c**). Yellow arrows point to PLK4 localization. **d** Distribution of PLK4 protein in relation to S phase reentry in dedifferentiated cells. All EdU+ dedifferentiated cells show centrosomal PLK4. Error bars represent 95% confidence intervals (Wilson method). (pooled culture plates, *n* = 8), Scale bars 10 μm.

Recruitment of PLK4 to centrioles is an important process for centrosome biogenesis, duplication and maintenance^32,41,42^. Therefore, we evaluated the spatial dynamics of PLK4 using our standardized mouse myoblast dedifferentiation protocol. In differentiated cultures, Cre-recombinase-induced tdTomato+ myotubes showed the expected cytoplasmic localization, whereas the residual mononucleates retained their canonical centrosomal PLK4 (Fig. S9). In myoseverin-treated cultures, the cytoplasmic PLK4 failed to relocalize to the centrosome when p53 was not inhibited (Fig. S10). In contrast, residual mononucleate cells, which represent the unfused mononucleate population, maintained PLK4+ centrosomal foci (Fig. S10). Importantly, when we inhibited p53 by Pifithrin-α, we found that dedifferentiated tdTomato+ mononucleate cells showed relocalization of PLK4 and formation of centrosomal structures (Fig. 5a–c). Among these cells, 40.4% showed EdU-incorporation (Fig. 5d). This agrees with the finding that not all mononucleate, myotube-derived progeny were centrin+ and γ-tubulin+ (Fig. S11) and that not all centrin+ and γ-tubulin+ cells were EdU+ (see, Fig. 3d). Importantly, all EdU+ dedifferentiated cells displayed centrosomal localization of PLK4 (Fig. 5d). These experiments show a link between the subcellular localization of PLK4 and the differentiated/dedifferentiated states of muscle cells.

### PLK4 in differentiated newt myofiber is essential for muscle dedifferentiation

**Fig. 6:**
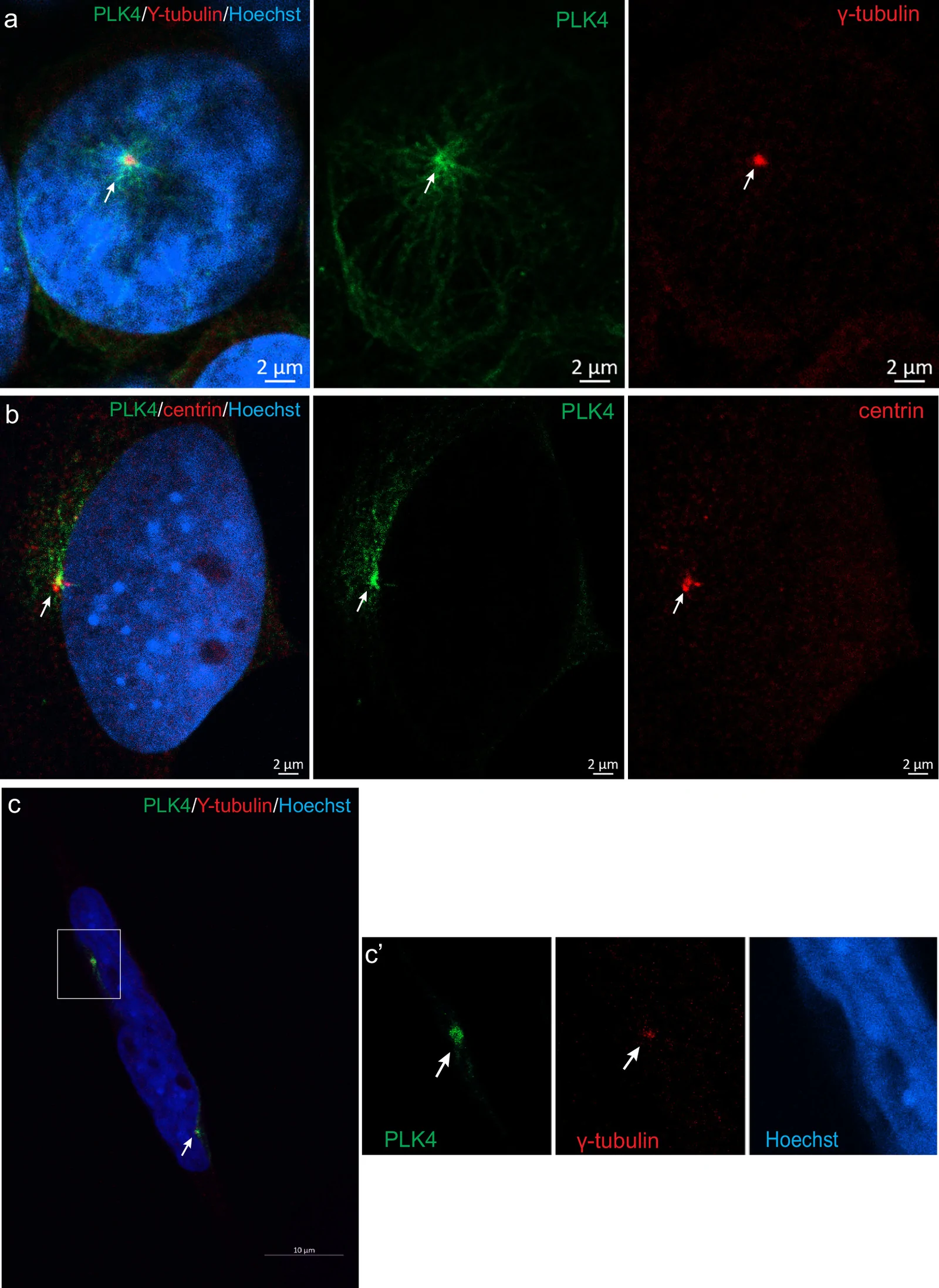
PLK4 colocalizes with other centrosome markers in newt cells. **a** Colocalization of PLK4 with γ-tubulin in a mononucleate cell. **b** Colocalization of PLK4 with centrin in a mononucleate cell. **c** Localization of PLK4 in centrosomes of a newt myofiber in culture. Colocalization with γ-tubulin is shown in inset (**c’**). In all panels, the white arrows point to centrosomes. Scale bars: **a**, **b** 2 μm; **c** 10 μm.

As we observed a redistribution of PLK4 during differentiation of mouse myoblasts, we next asked whether a similar shift in protein localization also occurred in newt primary myogenic cells and myofibers. We generated a custom peptide antibody against PLK4 (see Methods and Fig. S12). Immunofluorescence assay showed centrosomal colocalization of PLK4, based on both γ-tubulin and centrin reactivity (Fig. 6a, b) in mononucleate newt forelimb cells. Importantly, PLK4 was retained within centrosomes and showed colocalization with γ-tubulin also in isolated myofibers (Fig. 6c). Thus, in contrast to mice, muscle differentiation does not lead to intracellular reorganization of PLK4 protein in newts.

**Fig. 7:**
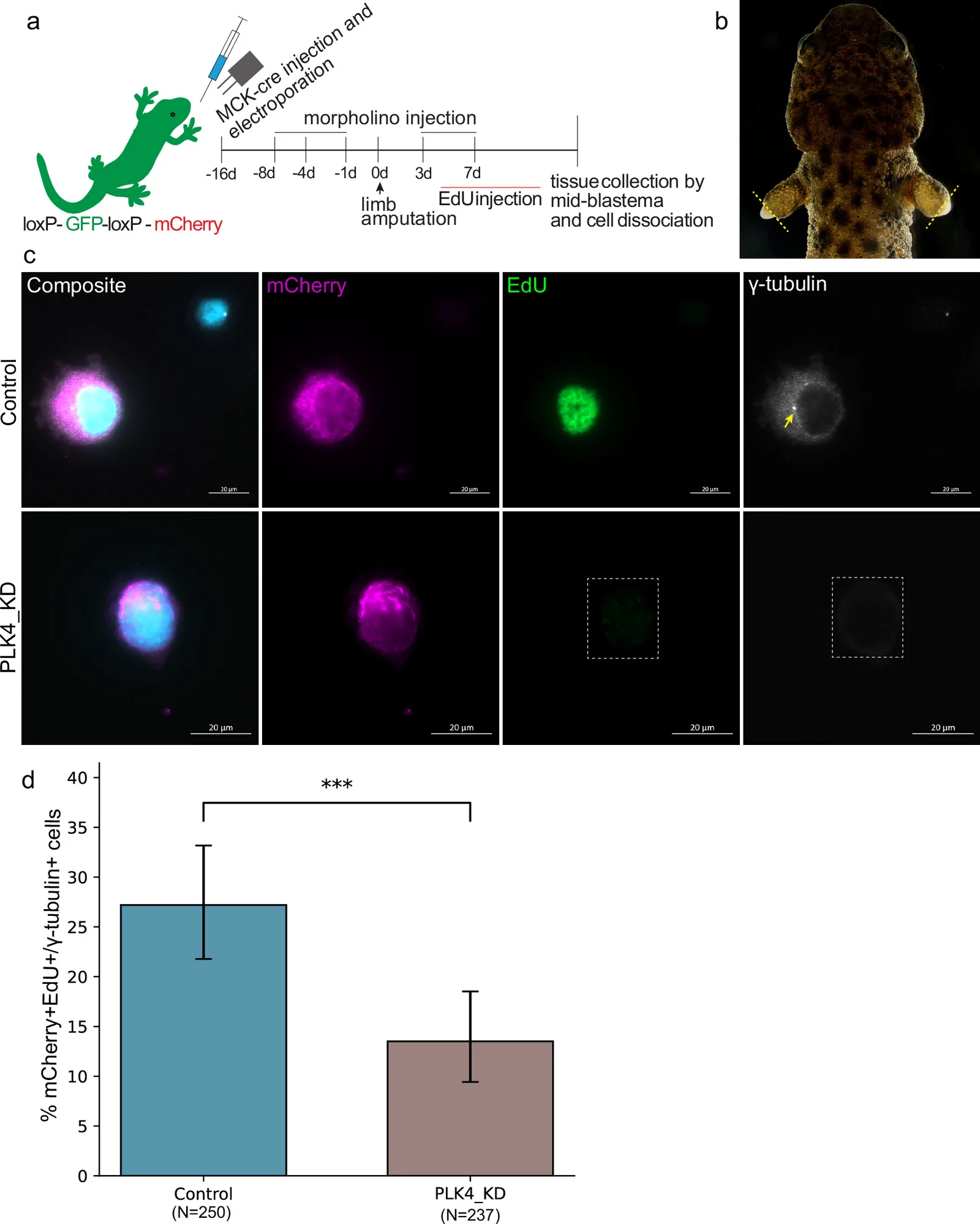
Depletion of PLK4 delays limb blastema formation and inhibits dedifferentiation. **a** Schematic showing the experimental outline. PLK4 morpholinos (PLK4_KD) were injected into transgenic newts to evaluate myofiber-progeny cell proliferation and blastema formation. **b** A representative adult newt with delayed limb blastema after PLK4 morpholino treatment (*n* = 8). Note the reduced blastema size (right limb) compared with the contralateral control morpholino-treated limb (left limb). **c** Example of a dedifferentiated muscle-derived progeny cell showing cell cycle reentry and centrosome formation indicated by γ-tubulin (yellow arrow, upper panel). Example of dedifferentiated myofiber-derived progeny cell isolated from PLK4 morpholino-treated limb blastema (lower panel). Note the absence of cell cycle reentry and lack of γ-tubulin staining (white dotted box. **d** Percentage distribution of dedifferentiated mCherry+/Edu+/γ-tubulin+ cells in the limb blastema after treatment with PLK4 morpholino (Two-sided Fisher exact test, 95% confidence level, *p* = 0.000199). Scale bars (**c**, **d**), 20 μm.

Stability of the centrosomes and retention of PLK4 protein in the newt myofibers prompted us to ask whether muscle dedifferentiation leading to proliferative mononucleate progeny requires PLK4. To test this, we used targeted custom PLK4 morpholino oligonucleotides in transgenic reporter newts (Figs. 7a and S13). Local introduction of PLK4 morpholino during regeneration led to substantial delay in blastema formation in five out of eight blastemas compared to pair-matched contralateral control limbs (Fig. 7b). To specifically assess the status of myofiber-derived dedifferentiated mononucleate cells, we dissociated morpholino-treated and control limb blastemas. Freshly dissociated cells were captured on EZ Megafunnel slides by Cytospin (see Methods) and processed for immunofluorescence. We detected mCherry^+^ cells from both control and morpholino-treated blastemas and assessed their S-phase reentry and centrosome status. This analysis showed a twofold reduction in blastema cells treated with PLK4 morpholino oligonucleotides (Fig. 7c, d). Some of the EdU− cells were also devoid of a specific centrosome moiety (Fig. 7c, lower panel; Fig. S13). Collectively, these results demonstrate that proliferation of myofiber-derived mononucleate progeny and blastema formation are dependent on the stability and retention of centrosomes and PLK4.

## Discussion

Centrosomes are critical cellular organelles for proper chromosome segregation during mitosis. Here we have shown that fully differentiated newt skeletal myofibers retain centrosomes that act as microtubule organizing centers. This is in sharp contrast to mammalian muscle cells, which gradually lose centrosomes during differentiation, and microtubule nucleation is organized from non-centrosomal localization^21,22,30,43^. Thus, retention of centrosomal microtubule organizing activity is a common feature shared between the multinucleate skeletal myofiber and the typically mononucleate cardiomyocyte in newts^34^. This common denominating feature gives a mechanistic explanation for why these two differentiated cell types retain their ability to give rise to proliferating progeny that subsequently contributes to tissue regeneration in newts.

Although a comparable dedifferentiation process does not occur in differentiated mammalian skeletal muscle cells that lost centrosomes naturally, we used here an experimental paradigm in which myotubes are forced into dedifferentiation, leading to cell cycle reentry and the generation of proliferative myotube-derived progeny. Since all analyzed EdU+ myotube-derived mononucleate cells had centrosomes, the data indicate that regaining proliferative potential requires centrosome reassembly. Although cell cycle reentry without centrosomes cannot be formally excluded, this may account only for a small fraction of the cases, if for any at all.

Centrosome dynamics have been linked to p53 activity. Increased p53 expression leads to downregulation of PLK4 and consequent failure in centrosome duplication^32,44^. Conversely, loss of p53 activity leads to centrosome multiplication^45–47^. In addition, it has been shown that knockdown of p53 allows cells to bypass cell cycle arrest at the G1-phase^40^. Our previous data on individually purified myotubes indicated that mammalian myonuclei are able to exit stable quiescence in the absence of p53 inhibition, but syncytial myonuclei do not reach S-phase^48^. The present results demonstrate that p53-inhibition leads to centrosome reassembly and further cell cycle progression, as shown by DNA replication.

How p53 is linked to centrosome reassembly in dedifferentiated muscle cells remains to be determined, but the shift in the subcellular localization of PLK4 gives an entry point to future investigations. Given that the expression level of PLK4 has been shown to be downregulated during cell differentiation in other cell types^39^, it was unexpected to find that PLK4 expression remained stable during myogenic differentiation. Since all EdU+ dedifferentiated mouse cells regained centrosomal localization of PLK4, this process represents a novel mechanism underlying the plasticity of the differentiated state in muscle. The importance of PLK4 localization is further underscored by the findings that its inhibition interferes with cell cycle reentry of dedifferentiated muscle cells during newt limb regeneration.

It is noteworthy that salamander limb regeneration involves downregulation of p53 activity, and limb regeneration is inhibited if p53 is stabilized^49^. Since we found that newt myofibers and their progeny in the blastema retain centrosomes, the function of p53 downregulation during limb regeneration should be other than allowing dedifferentiated muscle cells to proliferate without centrosomes.

Taken together, our cross-species comparative cellular assessment identifies a p53-mediated reorganization of centrosome components during cellular dedifferentiation of postmitotic skeletal muscle cells and highlights how the dynamics of centrosome components are linked to the plasticity of the differentiated state.

## Methods

### Animals and procedures

Animal experimentation was carried out and is described here according to the ARRIVE2 guidelines. Iberian newts (P. *waltl*) were captive-bred in our salamander facility in an environmentally controlled atmosphere according to Swedish animal welfare guidelines. Transgenic lines tgSceI(*CAG: loxP-GFP-loxP-Cherry*)^Simon^ and tgTol2(*CAG: loxP-Cherry-loxP-H2B:YFP*)^Simon^ were generated from wild-type newts. All animals were fed with live food or pellets. Larval animals of Stage 50-52 were used for experiments, whereas post-metamorphic newts were of age 6–8 months old. All animal procedures were carried out according to an approved Swedish ethical permit (permit # 3581-22, Karolinska Institutet), and post- operative care, including analgesics, was implemented in caring for the newts. The larvae were anesthetized in 0.05% Tricaine, while post-metamorphic newts were anesthetized in 0.1% Tricaine before surgical procedures. For the termination of experiments, the animals were anesthetized in the above medium for a prolonged time, decapitated, and brains pithed with sharp scissors as outlined in ethical permit 3581–2022.

### Primary culture of newt myofibers

Primary myofibers were isolated from forelimbs of larval P*. waltl* following the detailed protocol described previously^50^. The isolated muscle cell cultures were maintained in an incubator at 26 °C with 2.5% CO_2_ supply.

### Culture of newt cells

Newt cell cultures were derived from the forelimbs of adult *P. waltl* following the general guidelines described^50^ with modifications. Briefly, the limb tissues from zeugopodial and stylopodial regions devoid of skin were placed in dissociation medium (Collagenase I, 50 µg/ml; Dispase, 50 µg/mL; DNase I, 70 µg/mL; bovine serum albumin (BSA), Fraction V, 3.0 mg/mL; D-Glucose, 1.5 mg/mL)^50^ for 60 min in a shaking water bath at 26 °C, neutralized with serum-free amphibian MEM and filtered through 100 µm filter to remove the tissue debris. The filtrate was plated on a gelatin-coated culture plate, resulting in attachment and proliferation of cells. These cells were expanded and maintained as newt post-metamorphic limb cells. Cultures from passages 10 or above were used in experiments.

### Culture of mammalian cells

Pmi28 mouse myoblasts were originally obtained from Anna-Starzinsky-Powitz laboratory^30^. These cells were cultured on Matrigel-coated (1:10 dilution) flasks. Primary myoblasts were generated from Rosa26 transgenic mouse (*CAG:loxP-stop-loxP:tdTomato*) and obtained from Enric Llorens (Karolinska Institutet). Gastrocnemius muscles from the hindlimbs of a mouse were dissected, minced, and further dissociated in collagenase/dispase solution for 1 hour. The dissociation solution was neutralized in serum-containing medium, filtered through 40 µm filter, and the filtrate was pelleted, resuspended in serum-containing DMEM and plated on to 60 mm Petri dish. Adherent cells were expanded over generations, and passages above five were used in various assays.

### Microtubule regrowth assay

To analyze microtubule regrowth, the isolated myofibers were placed on ice for 3 h to induce depolymerization. Subsequently, the myofibers were returned to normal temperature to allow the recovery of the microtubules for 10–30 seconds. Before fixation, myofiber cultures were rinsed for 30 seconds at room temperature with 1% Triton-X-100 in PHEM buffer (60 mM PIPES, 25 mM HEPES, 10 mM EGTA, 2 mM MgCl_2_, pH 6.9). Thereafter the samples were fixed in pre-chilled Methanol at −20 °C, permeabilized with 0.5% Triton-X in PBS, blocked with 3% BSA in PBS for 1 h at room temperature and incubated with the relevant primary antibodies overnight at 4 °C. The next day, slides were washed in PBS and incubated with the corresponding secondary antibodies for 1 h at room temperature. Antibodies were diluted in blocking buffer, and sections were mounted in non-fluorescent mounting medium.

### Myotube cellularization assay

Rosa26 myoblasts (CAG:loxP-stop-loxP:tdomato) and Pmi28 myoblasts (MCK-Cre) were cultured separately in the presence of centrinone (5 µM) for four days. The cells were trypsinized and plated at a 3:1 ratio in a co-culture to induce differentiation in 2% horse serum-containing medium (low serum) in the presence of centrinone. After two days, centrinone was withdrawn from culture and cells were maintained in low-serum medium alone. On day 5, the cultures were treated with a p53 inhibitor, Pifithrin-α (60 µg/mL) for 24 h. On day 6, cellularization was induced in differentiated myotubes by treatment with myoseverin (25 µM) for 24 h. At 18 h during myoseverin treatment, the cultures were supplemented with Pifithrin-α and LIF (1000 U) to sustain p53 inhibition as well as to prevent fusion of dedifferentiated mononucleate cells. At 24 h after myoseverin treatment, the cultures were washed, and fresh growth medium containing programmed cell death inhibitors (DIDS, 100 µM; Q-VD 10 µM), growth factors (Pifithrin-α 60 µg/mL LIF, 1000 U; EGF 20 µg/mL; bFGF 50 ng/mL) and Thymidine 100 µM^51^ was added to the culture. In addition, EdU was incorporated (10 µM) to measure S-phase entry in cultured cells. After 24 h, the cultures were fixed and processed for immunofluorescence.

### Limb regeneration experiments

For the lineage-tracing of dedifferentiated muscle progeny cells, post-metamorphic transgenic newts (CAG:loxp-GFP-STOP-loxp-Cherry) and larval (CAG:loxp-Cherry-stop-loxp-H2B: YFP) transgenic newts were used. The right limbs were injected with a construct containing a muscle-specific promoter MCK, driving the expression of Cre-recombinase (~4 μg/μL), using a microinjector, while the contralateral limbs were served as un-injected negative controls. Immediately after injection, the limbs were electroporated using a Nepa21 Super Electroporator (Nepagene) with two pulses of 70 V for 35 ms each (poring pulse), followed by three pulses of 50 V for 1 s each (transfer pulse). In larval limbs, the voltages were two poring pulses of 35 V and six transfer pulses of 25 V at 50 ms intervals. The expression of the construct induced recombination in the myofibers, leading to the excision of the GFP gene and expression of the mCherry or H2BYFP reporter genes. We found that 10 days are sufficient for recombination events. Forelimbs were amputated at the mid-humerus level and allowed to regenerate for 10 days before they were collected. During this time, early dedifferentiation occurs from regenerating myofibers, and a good proportion of myofiber-derived progeny cells contribute to blastema formation. During the early blastema stage (~10 dpa), daily injections of EdU (0.1 mL/newt from a stock of 1 mg/mL) were given intraperitoneally until limbs were collected (at 12–16 dpa, depending on blastema size). The limbs were collected and fixed overnight in 4% formaldehyde at 4 °C, washed in PBS, cryoprotected in 20% sucrose, embedded in Tissue Tek O.C.T (Sakura) and frozen on dry ice. The samples were sectioned as 14 μm longitudinal sections in an NX70 cryostat (Thermofisher).

### Morpholino treatment

Standard control and *P. waltl* PLK4 morpholinos (Pw PLK4 e4i4: AGAAAGTTTGACTCACCTTCTTCTT and PwPLK4 e5i5: AATAATTATACTTACTGTGCATTTT) were designed and purchased from Gene Tools, LLC. The reagents were dissolved in distilled water as a 1 mM stock solution according to the manufacturer’s protocol. Transgenic reporter newts (CAG:loxp-GFP-STOP-loxp-Cherry) were used in the current experiment. To specifically label the myofiber, the muscle-specific MCK-Cre plasmid (2 µg/µL) was injected and electroporated into both forelimbs 16 days before limb amputation. Subsequently, morpholino oligos (a mix of equal volumes of Pw PLK4 e4i4 and PwPLK4 e5i5 with phenol red) were injected and electroporated into the right forelimb at specific intervals (see Fig. 7a). The contralateral left limbs were used as a control and received the control morpholino. After a series of morpholino injections, EdU was injected on 4 d post-amputation, and injections were continued on alternate days. Subsequently, the limb blastemas were harvested for cell dissociation and immunostaining.

### Blastema cell dissociation and cytospin

The PLK4 (right limb) and control (left limb) blastemas from different animals (*n* = 7) were collected and treated with 0.2% EDTA (in serum-free AMEM) in a water bath at 26 °C for 20 min. After careful removal of the wound epithelium, the tissues were transferred to the dissociation medium and incubated in a rocking water bath at 26 °C, with a speed of 90 rpm for 30 minutes. Following gentle trituration, the tissue suspension was returned, and dissociation continued at 60 rpm for 30 minutes. The dissociation was terminated by adding 2 mL of serum-free AMEM, and centrifuged at 200 × g for 10 minutes. The pellets were resuspended in 400 µL of AMEM. The EZ Single Megafunnel (Cat # A78710001, Fisher Scientific) slides were assembled based on the manufacturer’s instructions and 200 µl of the cell suspension was added into each cytospin funnel and centrifuged at 1500 rpm for 6 min with the low-speed settings. Subsequently, the samples were fixed with 4% buffered formaldehyde for 10 min, washed in PBS, post-fixed in methanol at −20 °C for 10 min, air dried and stored in airtight containers at −20 °C until use.

### Antibodies and immunofluorescence

Myoblast and myotube cultures were routinely fixed in 4% formaldehyde (Merck, cat #1004965000) for 10 min at room temperature, washed in PBS and post-fixed in 100% methanol at –20 °C for 10 min. Thereafter, the samples were briefly air-dried and washed in PBS containing 0.5% Triton-X-100 (PBST) and blocked in 3% BSA. For EdU labeling, the samples were processed according to the manufacturer’s protocol (Thermofisher). This was followed by antibody incubation of the samples with respective antibodies or in combination overnight at 4 °C. After primary antibody incubation, the samples were washed in PBST and incubated with secondary antibodies for one h at room temperature. The samples were washed again, counterstained the nuclei with Hoechst 33258, rinsed in PBS and mounted in Fluromount G.

For PLK4 antibody staining, the samples were fixed in a modified cytoskeletal buffer. The buffer solution was prepared by mixing 100 mM NaCl, 300 mM sucrose, 3 M MgCl_2_, 10 mM PIPES in sterile water, and the pH was adjusted to 6.9. To this solution 1 M EGTA and 0.5% Triton X-100 were added just before use. A 4% formaldehyde (from 16% FA: Thermofisher, EM grade) solution was prepared in cytoskeletal buffer to fix cell cultures for PLK4 staining. Before fixation, cultures were quickly rinsed in cytoskeletal buffer and subsequently fixed in the fixative for 15 min at room temperature. Processing for immunofluorescence was as described above.

For EdU labeling, Click-iT Plus EdU Imaging Kit was used throughout (Invitrogen, # C10640), following the manufacturer’s protocol. Cell cultures were incubated with EdU at 10 µM overnight in all cases, and the EdU labeling was performed before antibody labeling.

### Generation of newt PLK4 antibody

A custom peptide antibody to two non-overlapping regions of newt PLK4 protein (Peptide-1, ASAIGDKREDFKVLNLLGKGS; Peptide-2, DHRTAYQRDESDCSGRGRKA) was made in host chicken by David’s Biotechnologies, Germany. The reactivity of the antibodies was assessed in cultured *P. waltl* newt cells, and the optimal concentration was determined by titration. We used a modified extraction buffer (10 mM PIPES, 100 mM NaCl, 300 mM sucrose, 3 mM MgCl2, 100 mM EGTA, and 10% Triton X-100; pH adjusted to 6.9) containing 4% formaldehyde for the fixation of cell cultures. The cultures were initially rinsed in the extraction buffer for 30 seconds, and fixed for 10 min. Thereafter, cultures were washed in PBST buffer (Triton X-100, 0.25%) and post-fixed in ice -cold methanol for 10 min at -20 °C, washed again in PBST buffer, blocked in 3% BSA and processed for immunofluorescence. Of the two peptides, Peptide-1 gave specific reactivity with the PLK4 protein in newt cells, and a dilution of 1:20 was found to give optimal localization of PLK4 in centrosomes (Suppl. Fig. 12). Parallel incubation with concentration-matched non-specific antibody did not give any reactivity to centrosomes.

### Validation of PLK4 morpholino treatment

To validate the newt PLK4 morpholinos, Pw PLK4 e4i4 and PwPLK4 e5i5 were mixed in equal amounts and transfected into cultured newt cells. A control morpholino was transfected separately under the same conditions. Morpholinos (final concentration 10 µM) were mixed with Lipofectamine LTX reagent (Thermo Fisher Cat #15338030), and transfection was performed according to the manufacturer’s protocol.

The phenotype was evaluated 4 days post-transfection by immunostaining using a newt PLK4-specific antibody. In a parallel assay, the PLK4 knockdown effect was assessed by PCR. Total RNA was isolated from control and PLK4 morpholino-treated cells, and cDNA synthesis was performed using SuperScript IV reverse transcriptase (Invitrogen cat # 18090010) with Random Hexamers primer (Invitrogen cat # N8080127). The PLK4 frameshift induced by morpholino treatment was analyzed using the following gene-specific primers, Plk4 Forward 5’-GGAATGGTGCAACGAGTACG-3’ and Plk4 Reverse 5’-CTGGACAGCAGAAGGTTGG-3’.

### Microscopy and imaging

Muscle cell preparations were imaged under a Zeiss LSM 880 or LSM 900 microscope using ×63 Plan Apochromatic objective (N.A. 1.4 oil) and acquired using Zeiss ZEN software. In cellularization assays, the samples were imaged under a Zeiss Axio Imager Z1 or Axio Observer 7 microscope. The images were captured using a Hamamatsu Orca-Flash digital camera integrated with Zeiss ZEN software.

### Statistics

Statistical analyses and visualization were performed in Python using Scipy stats module or Graphpad Prism (Ver. 10.0). No randomization, sex differences or statistical assumptions were used in designing experiments or analyses. In all cases (*n*) represents the number of individual replicates in experiments, whereas (*N*) denotes total numbers.

***List of commercial antibodies:***

**Table:** 

Antibodies	Source	Cat number	Dilution	Purpose
Mouse anti-Ƴ-tubulin [GTU-88]	Abcam	ab11316	1:500	IF
Mouse anti-Ƴ-tubulin [GTU-88]	Sigma-Aldrich	T5326-25ul	1:500	IF
Rabbit anti-CEP135	Gift from Bettencourt Dias lab		1:1000	IF
Mouse anti-Centrin [20H5]	Sigma-Aldrich	04-1624	1:500	IF
Mouse anti-Sarcomeric α-actinin [EA-53]	Abcam	ab9465	1:500	IF
Mouse anti-MHC-GFP	Bio-techne	IC4470F	1:500	IF
Rabbit anti-RFP	Rockland	600-401-379	1:500	IF
Rat anti-mCherry	Invitrogen	M11217	1:500	IF
Mouse anti-mCherry	Nordic Biosite	677701	1:500	IF
Chicken anti-GFP	Sigma	AB16901	1:20	IF
Rat anti-α-tubulin [YL1/2]	Abcam	ab6160	1:500	IF
Rabbit anti-Myogenin	Thermofisher	14-5643-80	1:250	IF
Rabbit anti-PLK4	Nordic Biosite	AFT-5A49WW-100)	1:200	IF
Rabbit anti-MHC	Abcam	ab124205	1:500	IF
Mouse anti-MHC	DSHB	A4.1025	1:500	IF
Alexa Fluor 488 Goat anti-Mouse IgG	Invitrogen	A32723	1:500	IF
Alexa Fluor 647 Goat anti-Mouse IgG	Invitrogen	A21235	1:500	IF
Alexa Fluor 488 Goat anti-chicken Chicken IgY	Invitrogen	A-11039	1:500	IF
Alexa Fluor 594 Goat anti-Rabbit IgG	Invitrogen	A32740	1:500	IF
Alexa Fluor 488 Goat anti-Rabbit IgG	Invitrogen	A327731	1:500	IF
Alexa Fluor 555 Goat anti-Rabbit IgG	Invitrogen	A32732	1:500	IF

## Supplementary information

Subramanian et al. Figs. S1–14_Revised_260826

## Data Availability

No datasets were generated or analyzed during the current study.
