# Supplementary material for: Skeletal muscle dedifferentiation requires centrosomes
Source: NPJ Regen Med. 2026 Sep 25;11:38. doi: 10.1038/s41536-026-00509-3 (PMC13612385; doi:10.1038/s41536-026-00509-3)
Supplement: Supplementary file 1 [file 41536_2026_509_MOESM1_ESM.pdf]

Supplementary Information

Skeletal muscle dedifferentiation requires centrosomes

Elaiyaraja Subramanian, Anoop Kumar, Ketan Mishra, Gonçalo Brito, Matthew Kirkham and András Simon*

Department of Cell and Molecular Biology, Karolinska Institutet, Stockholm, Sweden

*Lead contact: andras.simon@ki.se

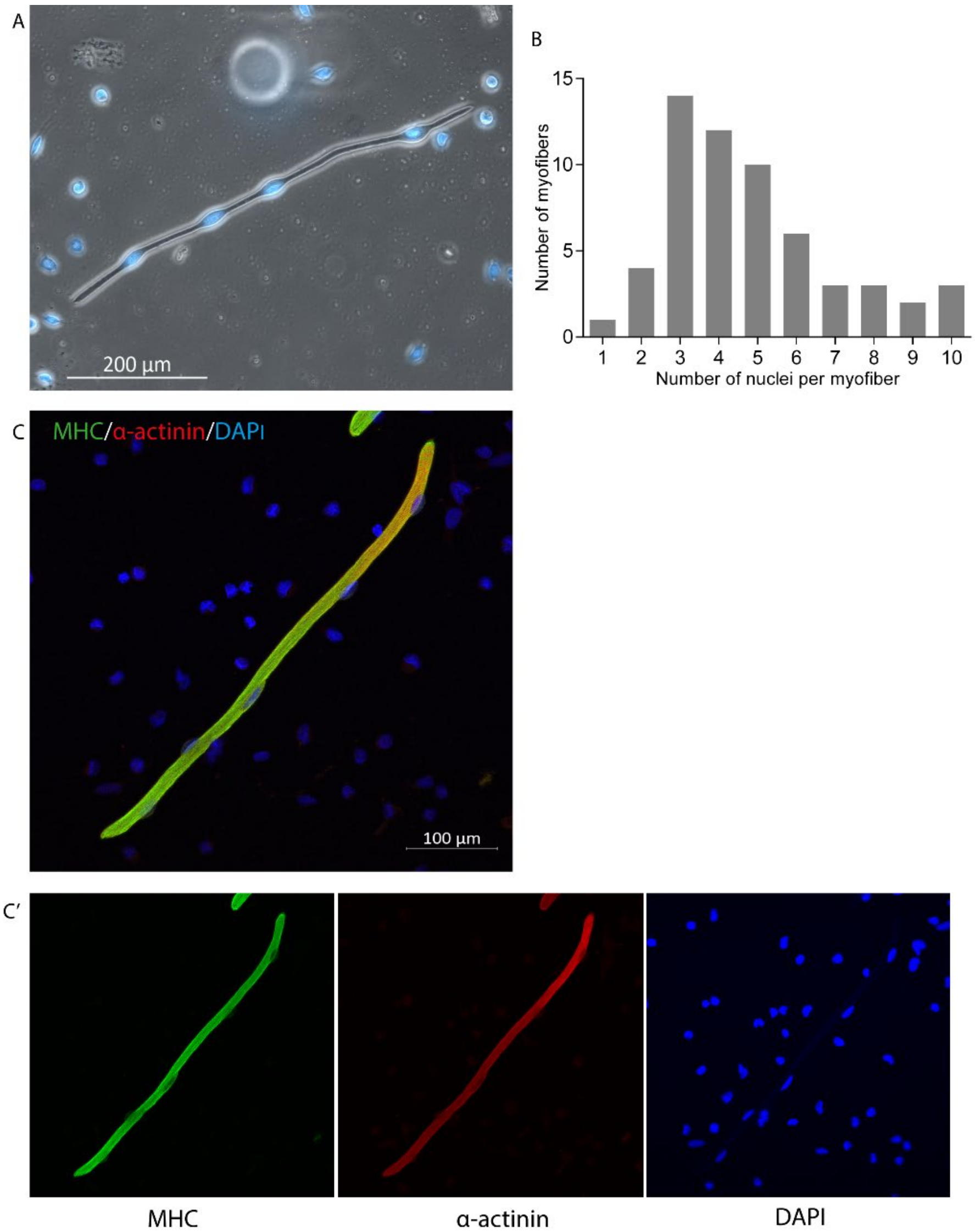

Fig. S1. **Characterization of newt myofibers.** (**A**) Live myofibers in culture incorporating the nuclear dye Hoechst 33342. (**B**) Distribution of nuclei in myofibers. (**C, C'**) Immunostaining of myofibers with muscle-specific antibodies to Myosin Heavy Chain (MHC) and α-actinin.

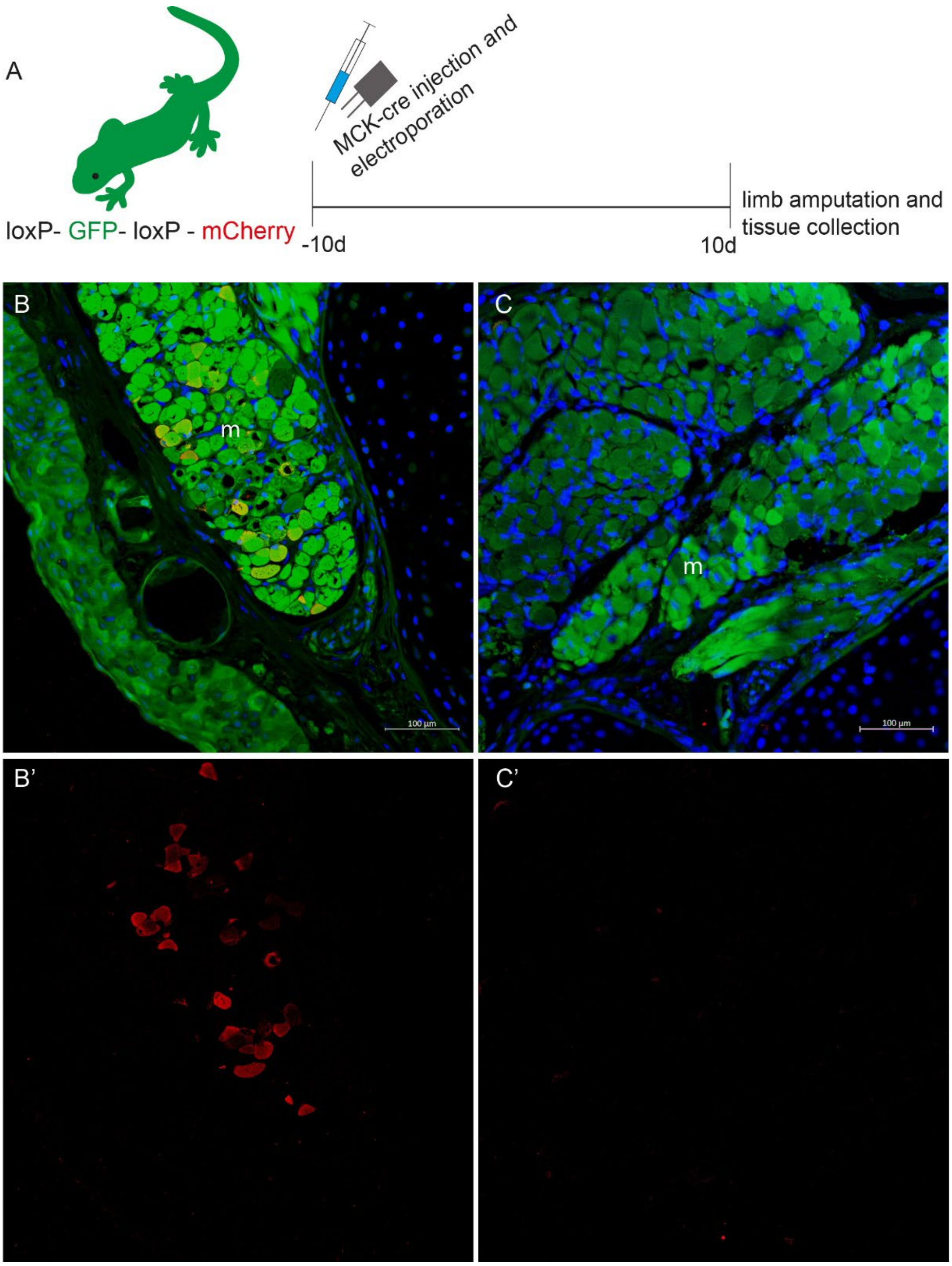

Fig. S2. Cre-induced recombination leads to mCherry expression in newt myofibers (**A**) Induction of MCK-Cre by electroporation in loxP-GFP-loxP-mCherry newt limbs. Focal electroporation leads to excision of GFP and expression of mCherry in muscle fibers. (**B**) Cross section of the newt limb 10d post-electroporation. (**B'**) The panel shows mCherry expression in muscle bundles. (**C**) Cross section of contralateral non-electroporated control limb. (**C'**) The panel shows the absence of muscle-specific mCherry expression. In all images, processing and contrast settings are applied equally. m: muscle bundles. Scale bars, 100 µm.

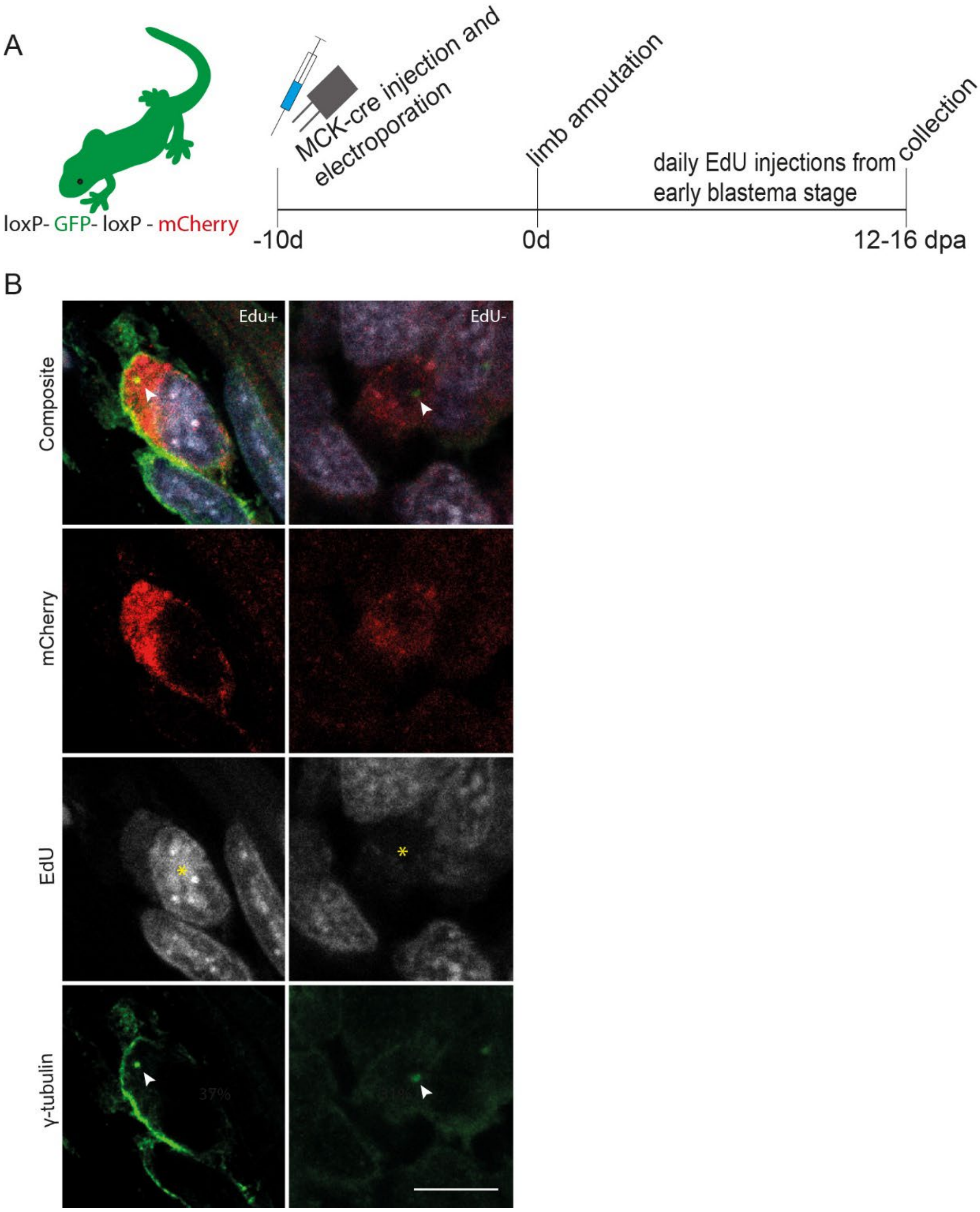

Fig. S3. **Centrosomes are retained by dedifferentiated mononucleate cells during newt limb regeneration.** (**A**) Outline of the experiment illustrating Cre-induced recombination leading to mCherry expression in myofibers. (**B**) Immunostaining on newt limb sections showing γ-tubulin+ centrosomes (arrowed white) in a dedifferentiated mononucleate cell (mCherry+/EdU+; left panel), and non-proliferating dedifferentiated muscle cells (mCherry+/EdU-; right panel). Yellow asterisk indicates EdU+ or EdU- cells in respective panels. n=8; Scale bar, 10 µm.

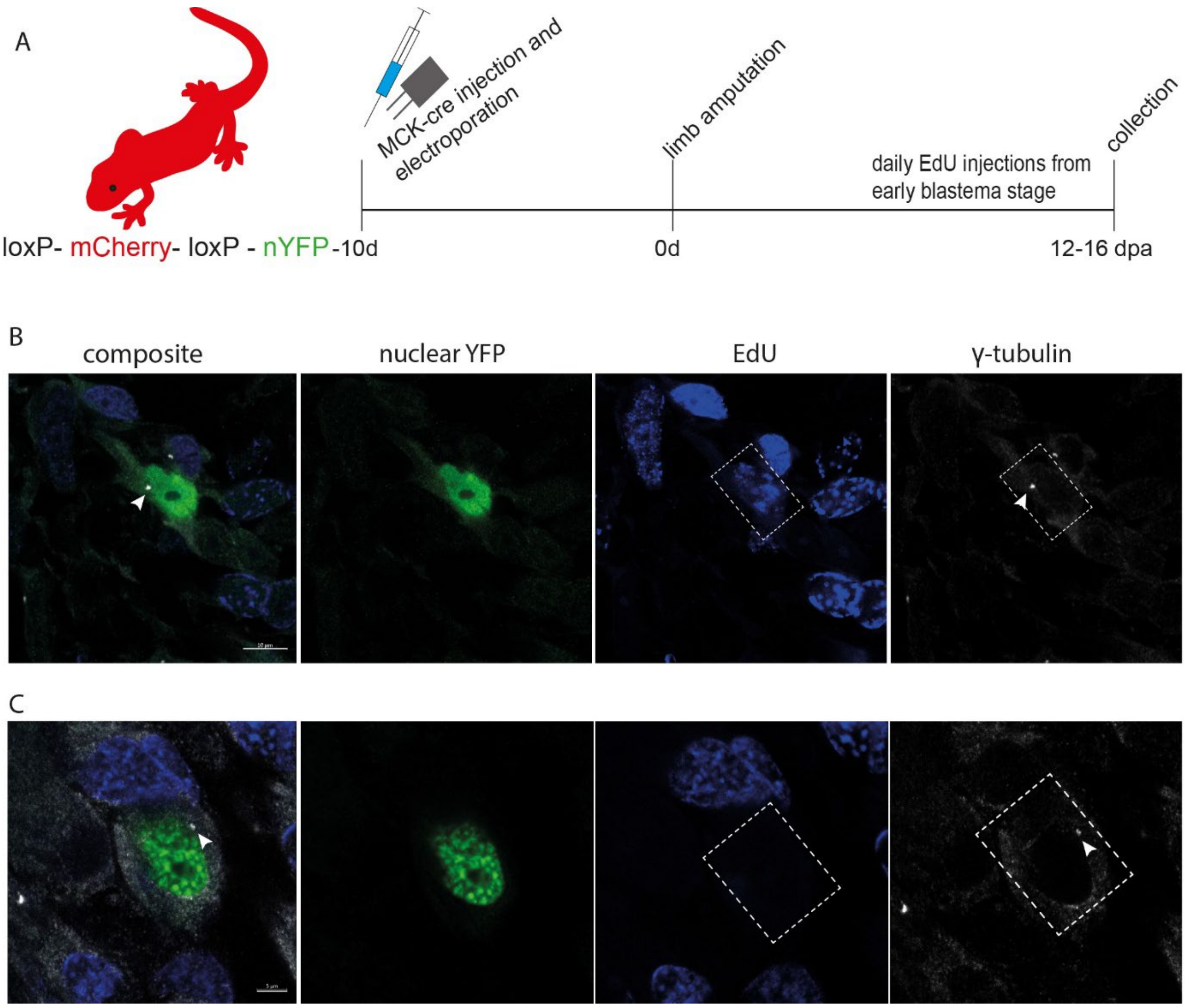

Fig. S4. Myofiber-derived mononucleate blastemal cells retain centrosomes. (**A**) Schematic illustration of MCK-Cre induction to induce nYFP expression in muscle fibers. (**B**) A representative image showing dedifferentiated mononucleate cells in limb sections. The cell has entered S phase as indexed by EdU (dotted white box). The centrosome within the cell is arrowed white. (**C**) A dedifferentiated mononucleate cell without S phase entry (dotted white box) showing presence of centrosome (arrowed white). Scale bars, 5 µm.

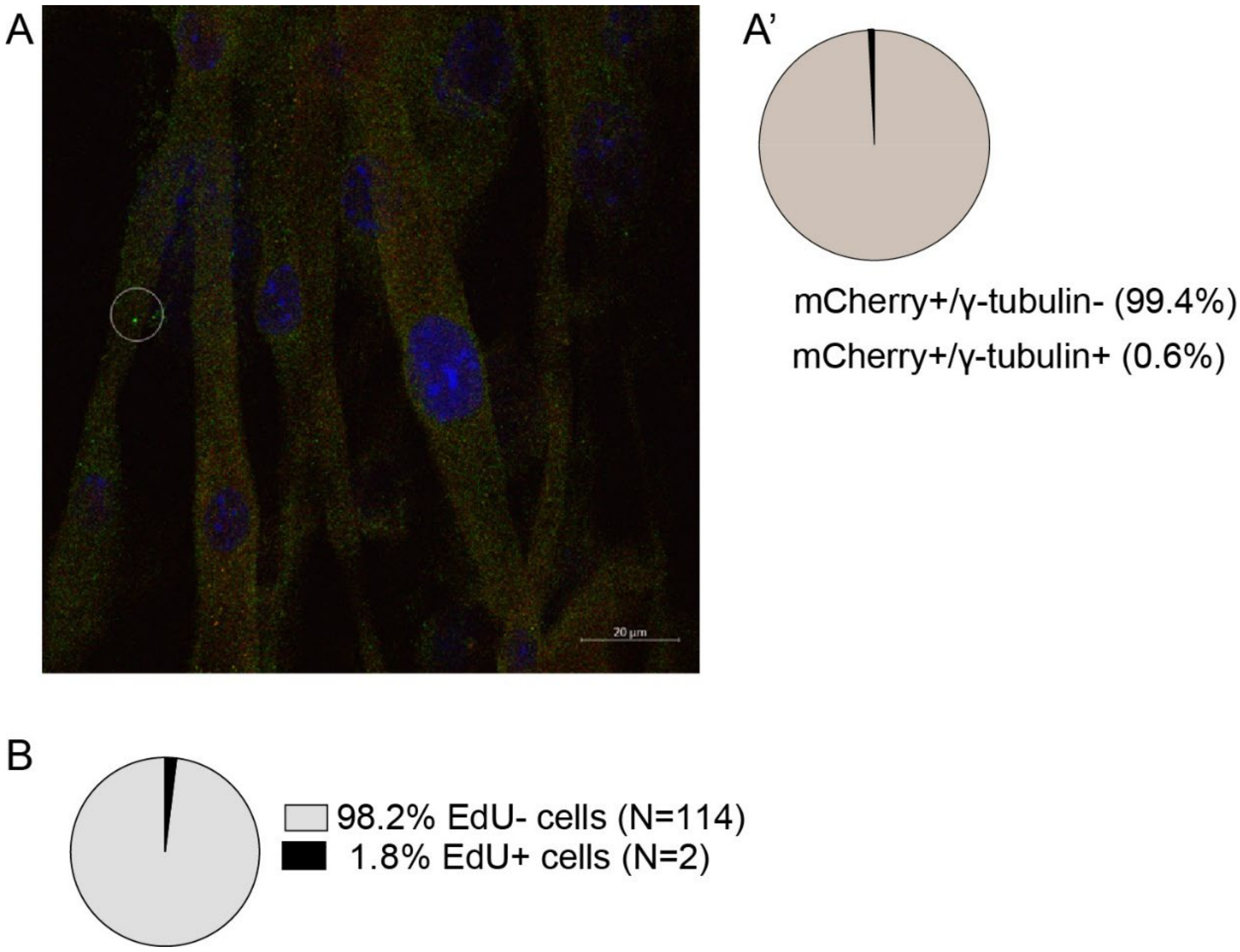

Fig. S5. **Depletion of residual centrosomes by centrinone-treatment and quantification of S-phase reentry without p53-inhibition.** (**A**) Representative image showing depletion of residual centrosomes from myotubes after centrinone-treatment. The myoblasts were treated with 5 µM centrinone for 4 days during myoblast culture and 2d during differentiation. The cultures were fixed and stained with antibody to mCherry and γ-tubulin. A residual γ-tubulin+ centrosome is circled in white. (**A'**) γ-tubulin staining shows lack of centrosomes in 99.4% of myotubes (N=181 myotube nuclei from 15 image fields). (**B**) Distribution of mononucleate cells in myotube cellularisation assay without p53 inhibitor (N=116 cells pooled from 8 culture plates).

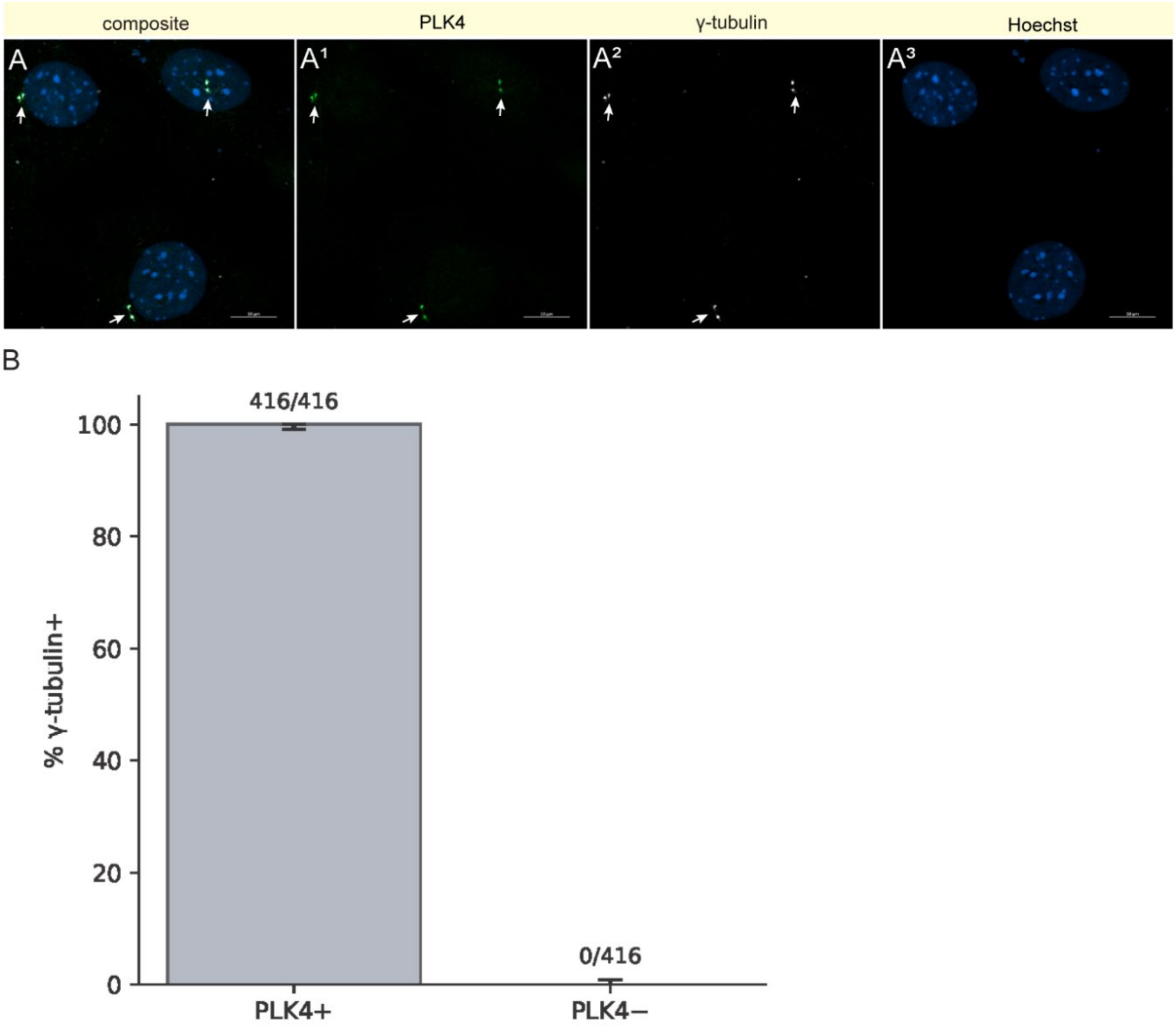

Fig. S6. **Validation of antibody against PLK4 in mouse myoblast cells**. (**A**) PLK4 protein is expressed in mouse myoblasts, and it is localized to centrosomes (arrowed white). Panels (A^1-A^3) represent split channels of the composite image. (**B**) Percentage distribution of γ-tubulin in PLK4+ myoblasts. Note the absence of any PLK4- cells. Error bars represent 95% confidence intervals (Wilson method). Total number of cells derived from three assays is indicated above the bar plot.

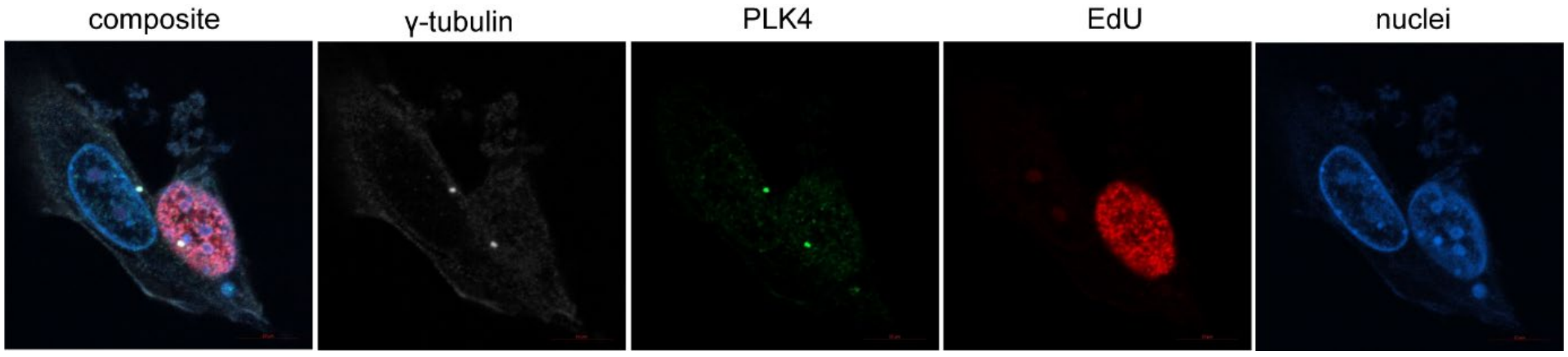

Fig. S7. **PLK4 localization is not cell cycle dependent in undifferentiated mouse myoblasts**. (**A**) Examples of cultured pmi28 myoblasts showing expression of PLK4 protein and its colocalization with γ-tubulin both in EdU+ and EdU- cells.

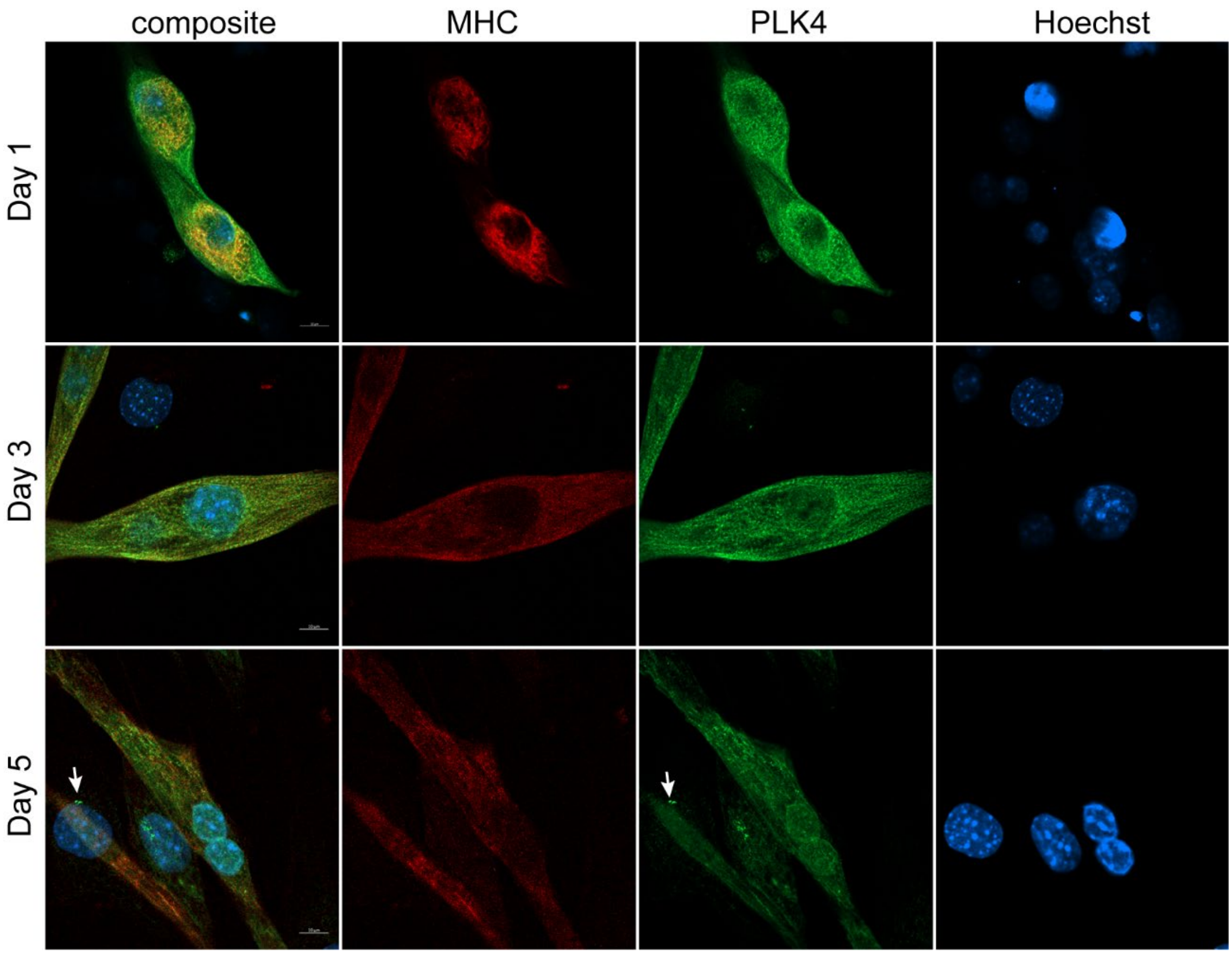

Fig. S8. PLK4 is redistributed in differentiated mouse muscle cells. Progressive differentiation of myoblast to form myotubes in culture. Note the cytoplasmic PLK4 expression in Myosin heavy chain (MHC)+ cells from Day1 to Day5. By day 3, the differentiating myotubes also show association of PLK4 and sarcomeres. Note the centrosome localization of PLK4 in unfused mononucleate cells in day 5 culture (arrowed white). Scale bars, 10 µm.

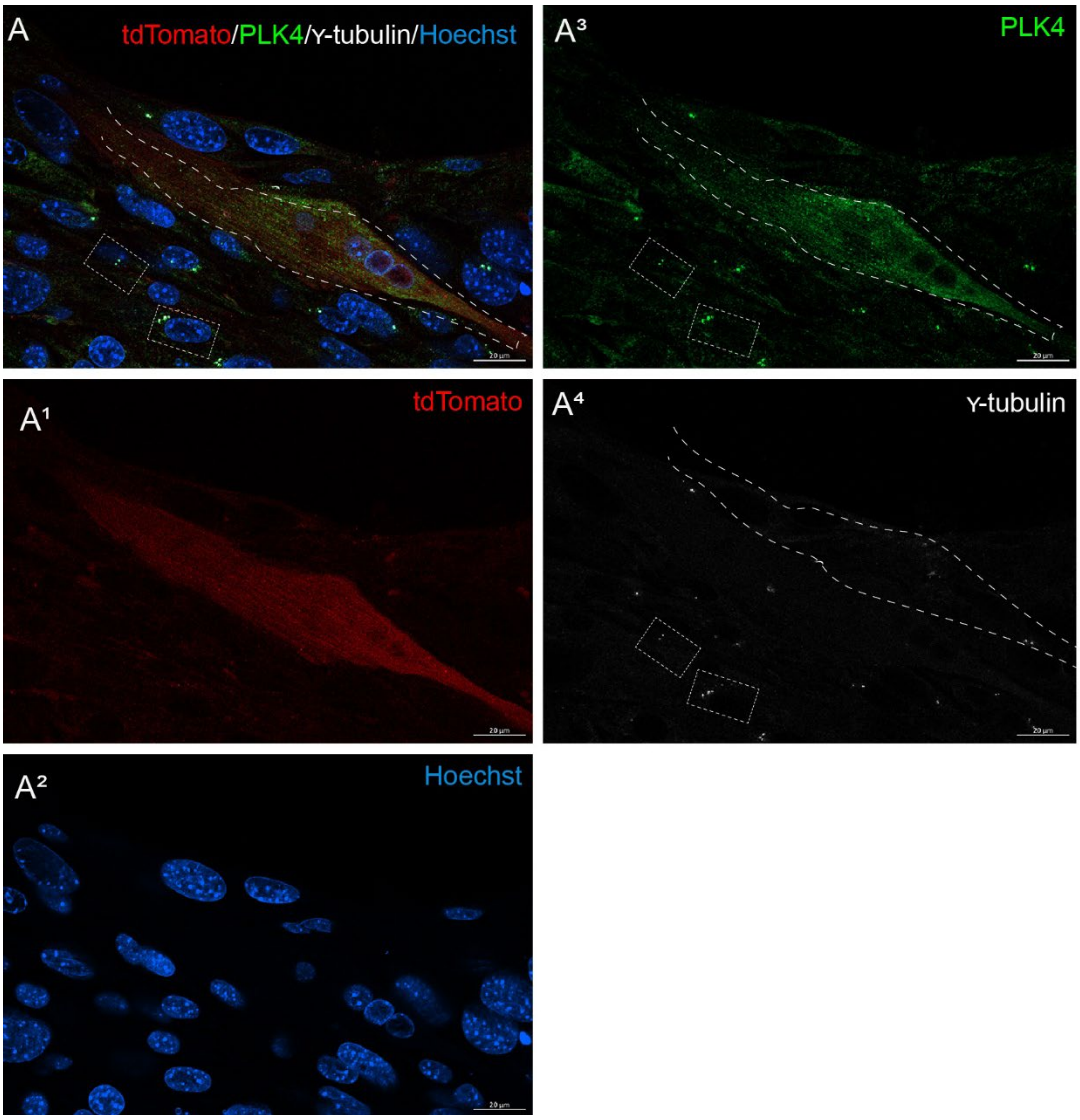

Fig. S9. Differentiated cultures of myotubes showing PLK4 dispersion in the cytoplasm without p53-inhibition. (**A**) A Cre-recombinase induced tdTomato+ myotube (outlined with dotted lines in (A), showing cytoplasmic localization of PLK4. Note the centrosomal localization of PLK4 in residual mononucleate cells (boxes with white dotted lines). (**A^1-A^4**) Split channels with respective markers. Scale bar, 20 µm.

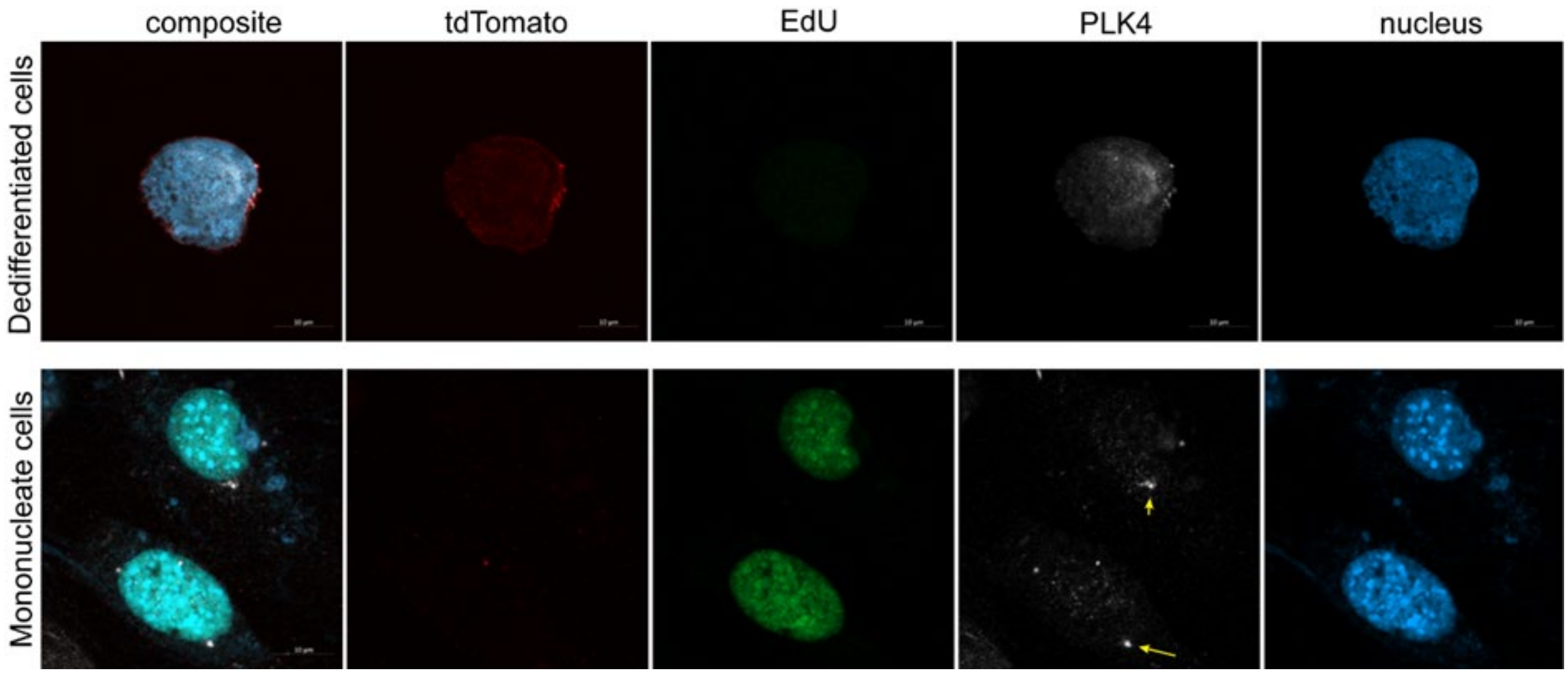

Fig. S10. **Lack of S-phase reentry and absence of centrosomal PLK4 localization in myotube-derived mononucleate cells without p53 inhibition**. A representative tdTomato+ mononucleate cell showing lack of S phase entry and centrosomal PLK4 (upper panel). Residual mononucleate cells from the culture show cell cycle reentry and PLK4 expression in centrosome (lower panel).

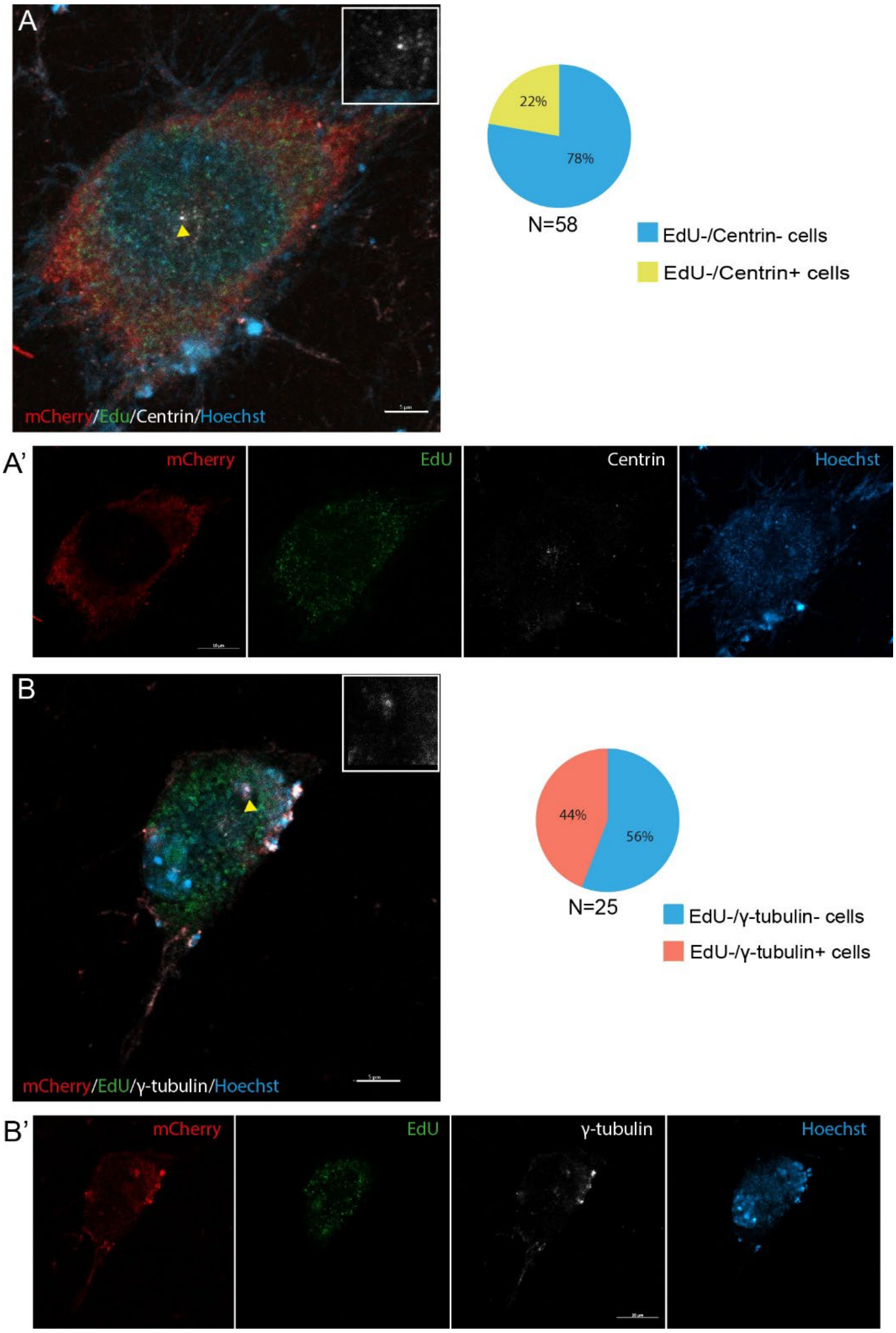

Fig. S11. **Relationship between cell cycle reentry and reassembly of centrosomes in myotube-derived mononucleate cells.** (**A**) Representative image showing mCherry+/EdU- cell with immunoreactivity for centrin (arrowed yellow). Inset: higher magnification of a centrosome, (**A´**) Split channels for the image shown in A. (**A´´**) Quantification of EdU$^-$ cells with centrin+ centrosomes in dedifferentiated cells (**B**) Representative image showing mCherry+/EdU- cell with reactivity for γ-tubulin (arrowed yellow). Inset: higher magnification of a centrosome. (**B´**) Split channels for the image shown in B. (**B´´**) Quantification of EdU$^-$ cells with γ-tubulin+ centrosomes in dedifferentiated cells. Scale bars, 5 μm.

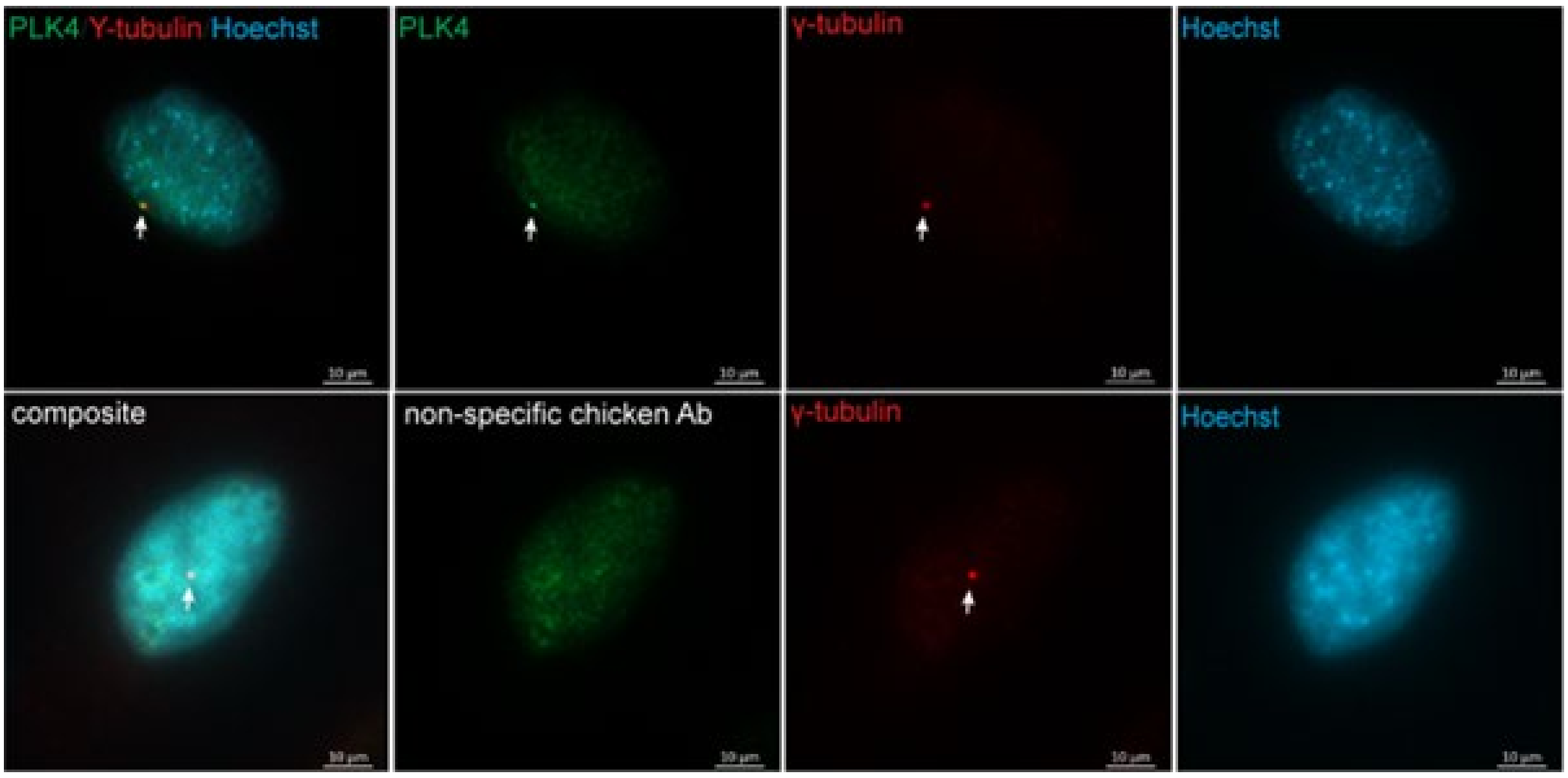

Fig. S12. **Evaluation of newt PLK4 antibody.** Custom peptide antibody to newt PLK4 was raised in chicken (see, Methods) and peptide antibody-1 was identified to specifically react with centrosomes (arrowed white) in cultured *P. waltl* forelimb cells (upper panel). A concentration-matched non-specific antibody was used in parallel assay which showed no reactivity with centrosomes in cultured *P. waltl* forelimb cells. The reactivity to γ-tubulin indicates the presence of centrosomes. Scale bar, 10 µm.

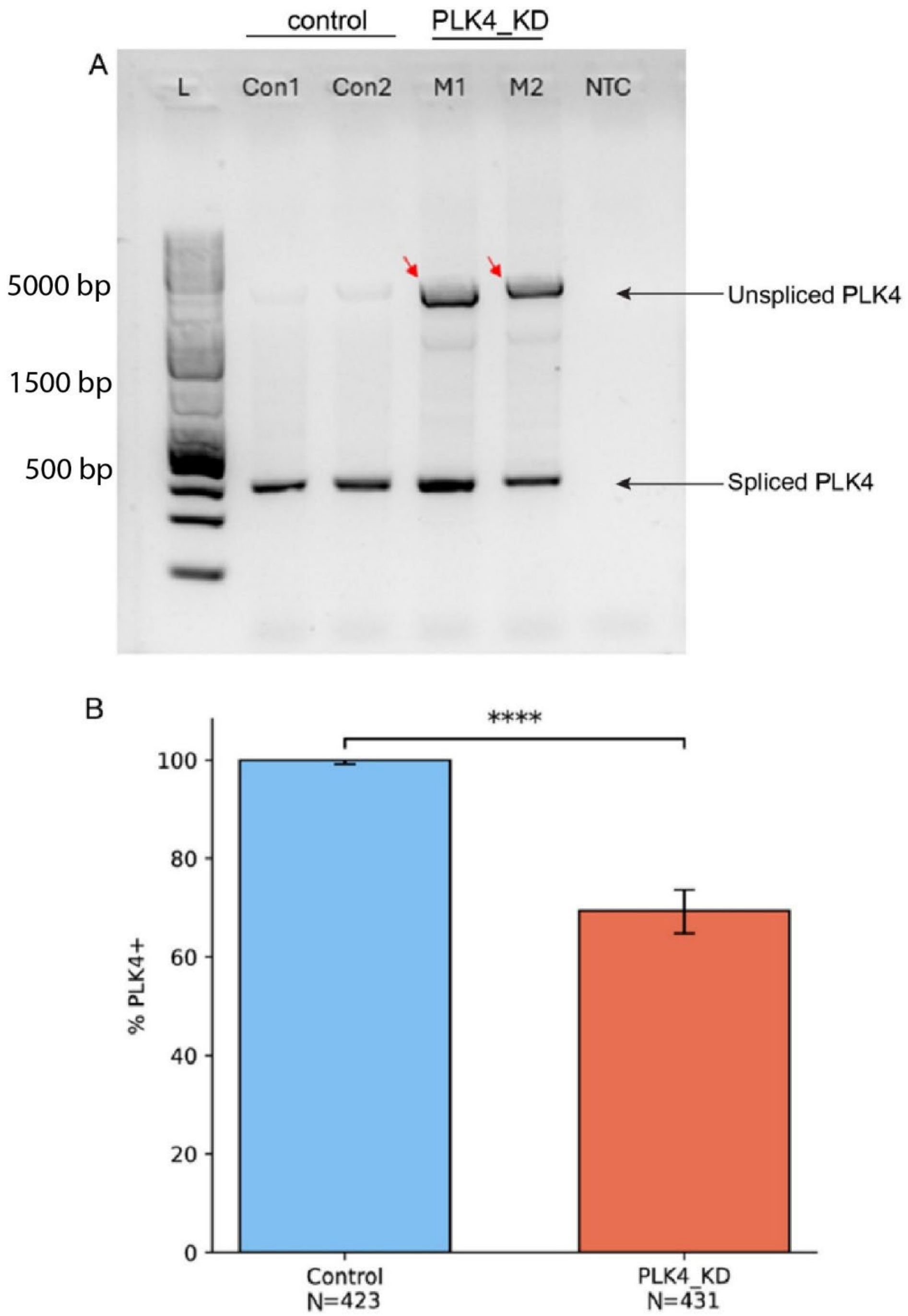

Fig. S13. **Validation of PLK4 morpholino in newt cells**. (**A**) Cultures of *P. waltl* forelimb cells were treated with 10 µM PLK4 morpholino (PLK4_KD) for 2 days. The PLK4 mRNA frameshift was analyzed using adjacent exon-targeted primers. Unspliced PLK4 band was observed only in the PLK4 morpholino-treated samples (arrowed red). Con1, Con2, control replicates; M1, M2, PLK4_KD replicates; NTC: non-template control. (**B**) The *P. waltl* forelimb cells were treated with 10 µM PLK4 morpholino for 4 days. The cultures were fixed and reacted with antibodies to newt-specific PLK4 and γ-tubulin. Percentage PLK+/- cells were evaluated from three replicates (Two-sided Fisher exact test, 95% confidence level; p = 4.99e-45 -> ****).

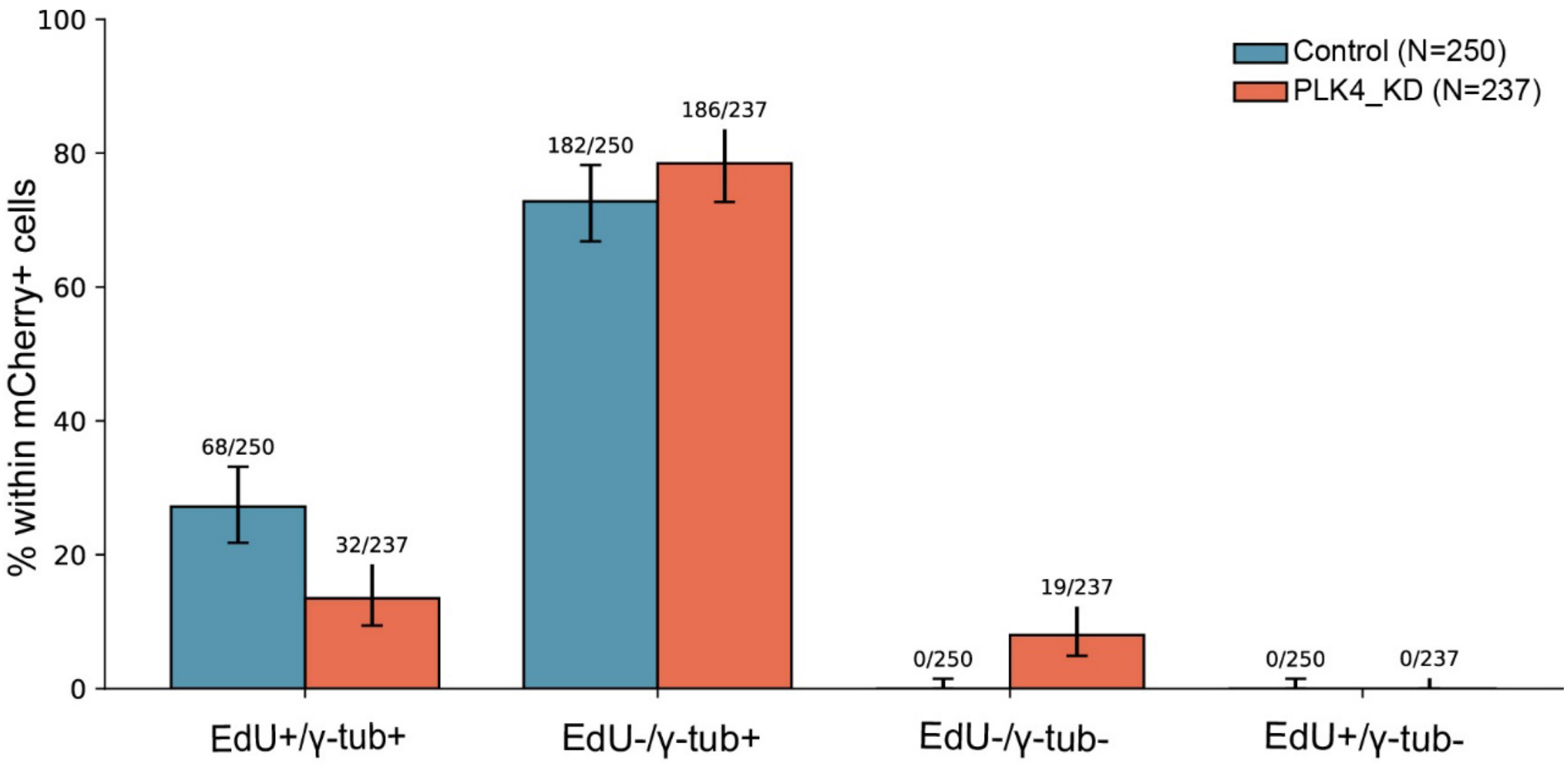

Fig. S14. Quantification of PLK4 knockdown and S-phase reentry in limb blastema. Distribution of mCherry+, myofiber-derived cells with or without S-phase reentry (EdU labelling) upon PLK4 knock down. γ-tubulin was used as a centrosome marker. Note the absence of γ-tubulin- cells within EdU+ cell populations. Error bars represent 95% confidence intervals using exact method.
